# Clusters, fingers, and singles: A mechanical landscape of tumor invasion

**DOI:** 10.1371/journal.pcbi.1014747

**Published:** 2026-09-08

**Authors:** Sheriff Akeeb, Adam I. Marcus, Yi Jiang

**Affiliations:** 1 Department of Mathematics and Statistics, Georgia State University, Atlanta, Georgia, United States of America; 2 Department of Hematology and Medical Oncology, Winship Cancer Institute, Emory School of Medicine, Atlanta, Georgia, United States of America; University of South Australia, AUSTRALIA

## Abstract

Collective invasion is a key mechanism by which tumors disseminate and metastasize, involving coordinated migration of heterogeneous cell populations. Experimental studies in spheroid-based assays have identified specialized leader and follower cells that work together during this process, but the biophysical rules governing their interaction remain unclear. We present a mechanistic, cell-based computational model using the Cellular Potts framework to investigate how heterotypic adhesion, leader motility, and follower proliferation jointly shape invasion. Leader–follower tumors were simulated across 13 310 parameter sets, and invasion was quantified by invasive and infiltrative areas, finger-like protrusions, solitary defectors, and detached clusters. From these simulations, we identified four distinct invasion phenotypes: non-invasive, bulk collective, single-cell, and multimodal. Multimodal invasion–the coexistence of cohesive strands, solitary cells, and small clusters–emerged as the most prevalent phenotype, particularly under moderate adhesion and high motility. Proliferation increased tumor bulk rather than determining invasion mode, which was governed primarily by adhesion and leader motility. Mapping outcomes across the parameter space revealed sharp transitions between invasion modes, underscoring trade-offs between adhesion and motility in shaping invasion complexity. Our results show that hybrid invasion behaviors, previously considered rare, arise robustly from simple mechanical rules and are favored in a broad region of the parameter space. This framework reconciles binary models of invasion with experimental observations of heterogeneity, providing predictive insights into how modulating adhesion and motility may modify invasive behavior.

## Author summary

When cancer cells leave a primary tumor, they do not just invade as lone cells but also as cohesive fingers and small cell clusters, depending on how strongly cells stick together, how hard some cells pull, and how fast the population grows. We built a simple, physics-based computational model in which a small number of “leader” cells pull on the more proliferative “followers.” By systematically varying three key properties, cell-cell adhesion, leader motility, and follower proliferation, we generated a large set of simulations of invasion patterns and grouped them into four invasion modes. The resulting phase diagram shows where tumors grow as compact masses (no invasion), or invade as protruding “fingers,” as single cells, or in a mixed multimodal fashion with fingers, clusters, and single cells. Surprisingly, this mixed state was the most common outcome, arising when adhesion is moderate and leader motility is strong. This map provides intuition and practical rules for thinking about how changes in adhesion or motility might reorganize invasion, for example, by shifting cluster-forming fronts toward more compact, less dissemination-prone configurations.

## Introduction

Metastasis, the leading cause of cancer-related mortality, often begins with local invasion, where tumor cells breach the basement membrane and infiltrate surrounding tissues. This process involves diverse cellular strategies that enable migration through complex microenvironments and ultimately facilitate colonization of distant sites [1,2]. A growing body of evidence distinguishes between two primary modes of invasion: solitary migration, where individual cells disseminate independently, and collective invasion, where groups of cells migrate in a coordinated, cohesive manner [3–5].

Solitary invasion is frequently associated with epithelial-to-mesenchymal transition (EMT), a process characterized by the loss of epithelial adhesion, upregulation of mesenchymal markers, and enhanced motility and invasiveness [5,6]. In contrast, collective invasion preserves cell–cell junctions and spatial organization, relying on mechanical coordination, intercellular signaling, and polarity among neighboring cells [7]. This mode of invasion is increasingly recognized across multiple carcinoma types, including breast [8], colorectal [9], and squamous cell cancers [10], and is associated with enhanced metastatic efficiency, immune evasion, and resistance to apoptosis [11].

A defining signature of collective invasion is the emergence of functional heterogeneity among migrating tumor cells, particularly the distinction between leader and follower subpopulations [12–15]. Leader cells, positioned at the invasive front, exhibit a constellation of specialized properties: high directional motility, robust chemotactic responsiveness, active proteolytic ECM remodeling through matrix metalloproteinases, formation of dynamic protrusions such as lamellipodia and invadopodia, and characteristically reduced proliferative activity [15,16]. These cells establish directional polarity, sense environmental gradients, and mechanically deform the surrounding matrix to create migration paths. In contrast, follower cells maintain strong intercellular adhesion through intact adherens and tight junctions, exhibit high proliferative capacity to replenish the migrating collective, and possess minimal intrinsic motility, instead moving passively through adhesive coupling to leaders and mechanical transmission of traction forces generated at the invasive front [17]. This functional dichotomy creates a symbiotic relationship: leaders provide directional guidance and path generation, while followers contribute structural cohesion and biomass expansion. The division of labor mirrors developmental migratory systems, most notably tip-stalk cell organization during angiogenic sprouting, where tip cells guide vessel outgrowth while stalk cells proliferate to form the nascent vessel tube, and neural crest cell migration, where leader cells blaze trails while followers maintain collective cohesion [16].

Recent experimental advances, including the Spatiotemporal Genomic and Cellular Analysis (SaGA) platform, have enabled the molecular profiling of leader and follower cells, revealing transcriptional and phenotypic signatures that confirm their distinct roles in invasion [12,18]. These findings underscore the importance of spatial heterogeneity within tumors and raise critical questions about how biophysical properties such as cell–cell adhesion, motility, and proliferation give rise to diverse invasion behaviors.

Despite these insights, most computational models of cancer invasion simplify the invasion spectrum into binary categories, collective or solitary, and often neglect the spatial, mechanical, and phenotypic dynamics observed in real tumors [17,19–22]. Prior theoretical frameworks have provided important foundations: Szabó and colleagues identified density-dependent phase transitions in collective tissue cell migration [23], while continuum reaction-diffusion models by Sherratt and colleagues revealed how traveling wave dynamics shape invasion fronts [24–26]. Turner and Sherratt extended these insights using the Cellular Potts Model to demonstrate that intercellular adhesion strength determines invasion morphology [27]. More recently, experimental and computational work by Bhat and colleagues has shown that reaction-diffusion dynamics coupled with cell-matrix adhesion generate fingering instabilities and multiscale invasion patterns in tumor spheroids, with cluster phenotypes correlating with clinical outcomes [28–30]. However, few models explicitly represent leader–follower heterogeneity or systematically explore how interactions among adhesion, motility, and proliferation shape invasion phenotypes across comprehensive parameter landscapes. As a result, existing frameworks often fail to capture the complex, hybrid behaviors increasingly observed in aggressive cancers, such as the coexistence of collective strands, solitary cells, and detached clusters within a single tumor front [31].

To address this gap and isolate the fundamental mechanical interactions governing collective cell migration, we develop a biophysical, cell-based computational framework that represents a simplified in vitro invasion assay rather than the full complexity of in vivo tissue invasion. Using the Cellular Potts Model (CPM) [32,33], we explicitly incorporate leader–follower dynamics within a two-dimensional spatial domain representing spheroid-based invasion assays. This idealized model abstracts away basement membrane architecture, organ tissue context, and stromal microenvironment, allowing us to systematically explore how cell-cell adhesion, motility, and proliferation interact to shape invasion behaviors in controlled experimental settings. We simulate over 1,331 unique parameter combinations, each with 10 replicates, to explore the emergent invasion behaviors.

We hypothesize that the interplay among adhesion, motility, and proliferation governs transitions between distinct invasion modes, and that multimodal invasion, characterized by simultaneous collective strands, single-cell dispersal, and detached clusters, arises robustly under specific biophysical regimes. Our goal is to construct a high-resolution phenotype map that links local cell-level properties to tissue-scale invasion strategies, providing both mechanistic insight and predictive capacity.

By integrating mechanistic modeling with data-driven classification, our study advances the theoretical understanding of collective invasion and leader–follower coordination, offering a quantitative framework for interpreting tumor heterogeneity and potentially informing therapeutic strategies to disrupt metastatic dissemination.

## Model and methods

### Computational model of cancer invasion

To study the collective invasion of solid tumors, we developed an in silico model using the software CompuCell3D (CompuCell3D.org) version 4.6.0 [34], a modeling environment implementing the lattice-based Cellular Potts Model (CPM) framework [32,33,35]. The CPM is a stochastic, energy-minimizing framework for simulating multicellular systems in which cells are represented as extended, deformable domains on a discrete lattice. The mathematical formulation of the CPM energy functional and the Metropolis algorithm governing system dynamics are presented below. The total energy of the system is given by:


$$\begin{array}{c} H=\sum_{\langle i,j\rangle }J\left(\tau (\sigma_{i}),\tau (\sigma_{j})\right)(1-\delta_{\sigma_{i},\sigma_{j}})+\sum_{\sigma }\lambda_{V}(V_{\sigma }-V_{\sigma }^{T}{)}^{2}-\sum_{\vec{x}}\lambda \cdot c(\vec{x})\cdot \delta_{\sigma (\vec{x}),\text{LC}} \end{array}$$ (1)

The first term captures cell–cell and cell–medium interactions through a contact energy matrix $J(\tau ,\tau^{\prime })$, where τ(σ) denotes the type of the cell occupying lattice site σ. The Kronecker delta function $\delta_{\sigma_{i},\sigma_{j}}$ ensures that this energy contribution only arises at interfaces between distinct cells, thus quantifying interfacial tension due to differential adhesion. Lower values of *J* correspond to stronger adhesion, while higher values indicate weaker adhesive interactions. The second term imposes a volume constraint on each cell. $V_{\sigma }$ is the current volume of cell σ, and $V_{\sigma }^{T}$ is its prescribed target volume. Deviations from the target are penalized with strength $\lambda_{V}$, thereby enforcing volume conservation and preventing biologically unrealistic cell expansion or shrinkage. This constraint is crucial in capturing the physical limitation of space and mechanical pressure within dense tumor tissues. The third term encodes the migratory responsiveness of leader cells. $c(\vec{x})$ is the local chemoattractant concentration at position $\vec{x}$, and λ is the migratory sensitivity coefficient that quantifies how strongly leader cells respond to gradients in the chemical field. The Dirac delta function $\delta_{\sigma (\vec{x}),\text{LC}}$ restricts this term to only those lattice sites occupied by leader cells, enabling directed migration along chemoattractant gradients. In this model, follower cells are non-migratory and experience only passive motion through adhesion and growth [12].

Cell configurations evolve stochastically through attempted lattice updates using the Metropolis algorithm, where each attempted cell ID copy from site $\vec{x}$ to a neighboring site ${\vec{x}}^{\prime }$ is accepted with a probability governed by the Boltzmann distribution:


$$\begin{array}{c} P(\Delta H)=\left\{ \begin{array}{cc} 1 & \;\text{if }\;\Delta H\leq 0\\ e^{-\Delta H/T} & \;\text{if }\;\Delta H>0 \end{array}\right. \end{array}$$ (2)

where ΔH is the change in energy associated with a proposed lattice update, and *T* is the effective simulation temperature controlling membrane fluctuations or biological noise. One Monte Carlo Step (MCS) comprises a number of attempted lattice-site copy events equal to the total number of lattice sites, so that each lattice site is selected once on average. Simulations were run for 700 MCS, corresponding to approximately 36 h of biological time. This framework was implemented using CompuCell3D (version 4.6.0), a modular simulation environment for CPM that incorporates PDE solvers, cell tracking modules, and customizable cell behaviors, such as chemotaxis and volume regulation [34].

### Tumor domain, spatiotemporal calibration, and simulation parameters

The computational tumor represents key features of non-small cell lung cancer (NSCLC) tumor spheroids used in experimental systems such as the Spatiotemporal Genomic and Cellular Analysis (SaGA) platform [12].

**Fig 1:**
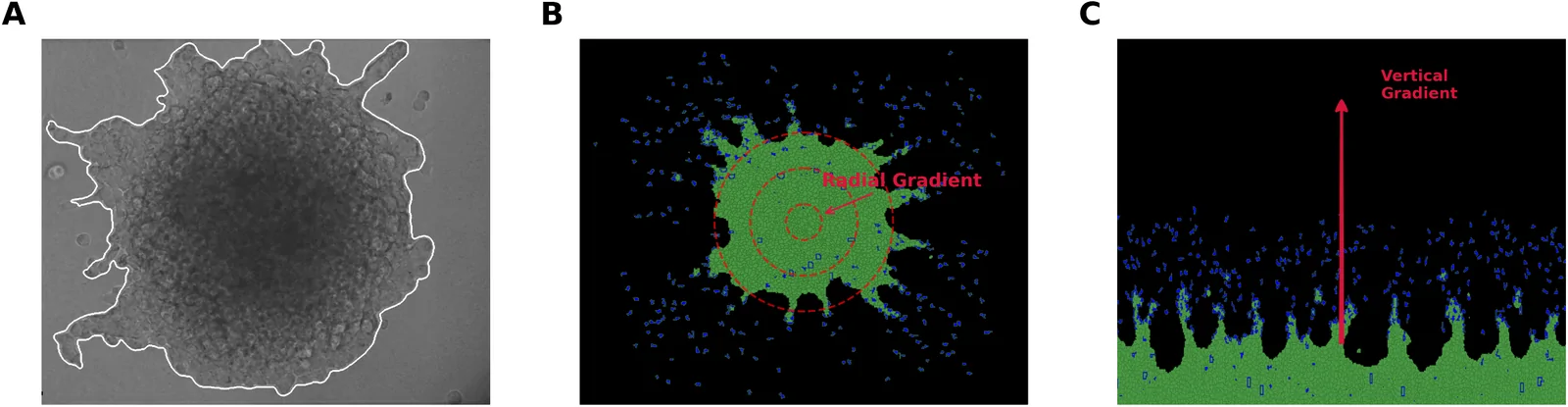
Geometry abstraction from experimental spheroids to in silico domains. **(A)** Experimental tumor spheroid embedded in ECM with leader-driven finger-like protrusions at the invasive front. **(B)** In silico spheroid under a radial migratory field; dashed concentric rings indicate radial distances, and the “Radial Gradient” annotation marks outward bias. Leader cells (blue) respond chemotactically to the gradient and drive directional invasion, while follower cells (green) proliferate and maintain tumor integrity through cell-cell adhesion. **(C)** Rectangular slab abstraction used in this study. Cells are initialized at the base with leader cells (blue, 25% of population) possessing a high migration coefficient (λ) and follower cells (green, 75% of population) exhibiting a high proliferative probability (*PP*). All cells are initialized at the base and exposed to a vertical stimulus that increases along the y-axis (arrow), with only leader cells responding through directed migration while follower cells move passively through mechanical coupling to the leaders. This reduced 2D geometry preserves directional invasion behavior and leader-follower dynamics while enabling reproducible, high-throughput parameter sweeps. S1 Video illustrates the temporal evolution of the corresponding 3D simulation, confirming that the reduced 2D slab abstraction effectively captures the key structural features of tumor invasion.

This simplified model represents an idealized in vitro invasion assay without explicit representation of a basement membrane, organ tissue context, or stromal microenvironment elements present in vivo, but secondary to the mechanical cell-cell interactions that are the focus of this study. While tumor invasion typically begins as 3D spheroids embedded in extracellular matrix (ECM), we adopted a quasi-two-dimensional cross-section abstraction representative of experimental invasion assays used in experimental oncology, where spheroids are embedded in Matrigel or collagen, discretized on a 500×300 lattice (≈1,250×750μm) with periodic boundary conditions along the horizontal (x) direction, as illustrated in Fig 1. This domain size was chosen to accommodate invasive fronts extending up to 500 micrometers from the initial tumor while maintaining computational tractability for large-scale parameter sweeps.

In adopting this 2D abstraction, we made three simplifying assumptions. First, 2D simulations preserve key 3D invasion dynamics: prior CPM studies [36–38] have demonstrated that 2D cross-sections retain essential morphological features, including finger formation, growth dynamics, and spatial patterning, in 3D tumor models. Our preliminary 3D spheroid simulations (S1 Fig, S1 Video) confirmed that invasive area, finger counts, and cluster incidence differed by less than 15% between 2D and 3D under equivalent parameter sets. Second, ECM remodeling does not qualitatively alter invasion modes within the simulation timeframe. We have previously quantified collagen fiber alignment as a consequence of cancer cell invasion over 24-hour periods [39,40], finding that significant matrix reorganization occurs on timescales of 24–48 hours. Since our simulations span approximately 36 hours (700 MCS), the static ECM assumption is reasonable for capturing initial invasion dynamics before substantial matrix feedback becomes dominant. Third, the tumor-front curvature inherent to spheroid geometry does not fundamentally alter the parameter dependence of invasion phenotypes. While absolute metric values may differ between planar and curved fronts, the transitions between no invasion, single-cell, bulk collective, and multimodal invasion are governed primarily by the balance between adhesion and motility, which is preserved in the 2D slab geometry.

The model consisted of two biologically distinct cell types: leader cells (LCs), which exhibit strong directed migration, and follower cells (FCs), which proliferate stochastically but lack intrinsic motility. We initialized the simulated tumor as a planar slab of follower cells (FCs) occupying the bottom of the domain, spanning the full width of 500 pixels and a height of 21 pixels (*y* = 0–21), the latter corresponding to approximately 52.5 µm. This configuration contained approximately 1167 follower cells, with exact counts varying slightly due to stochastic initialization. A random subset of 25% of these cells was then reassigned as leader cells (LCs), consistent with experimental observations of leader fractions of approximately 30% in NSCLC spheroids [41]. To confirm that this choice does not qualitatively alter the findings, simulations were additionally performed at leader fractions of 15% and 35%, bracketing the chosen value. The spatial structure of all five invasion metric landscapes and their parameter sensitivities were preserved across all three conditions (S9 Fig), supporting the robustness of the 25% condition as a representative value. This configuration mimics the basal cross-section of a spheroid tumor embedded in the ECM, thereby maintaining the invasion polarity and architecture observed in vitro.

A spatially static, non-diffusing chemoattractant gradient field $c(\vec{x})$ (implemented as a CompuCell3D chemical species named ’MV’ for migration vector) was imposed along the y-axis to simulate a constant directional migration cue. The gradient was defined as *c*(*x*,*y*) = *y*/*g*, where *g* = 1 sets the *g*radient stren*g*th. This linear gradient represents a fixed directional stimulus consistent with vertical invasion observed in Matrigel-based assays [42], and could correspond biologically to oxygen gradients, growth factor gradients, or other chemotactic cues that guide leader cell migration away from the tumor core. This quasi-2D slab configuration preserves essential directional invasion dynamics while reducing computational complexity, enabling systematic exploration of key biophysical parameters across more than 13,000 simulations.

The correspondence between simulation spatiotemporal scales and in vitro tumor cell behavior was established by calibrating leader cell migration speeds to experimentally measured values from 3D collagen-based invasion assays [12]. Leader cells exhibited mean velocities of (1.1 ± 0.3) µm/min. We measured leader cell displacement at λ=20 (intermediate motility) and obtained 0.4 px/MCS. Setting the spatial scale to 1 px ≈ 2.5 µm (guided by typical carcinoma cell diameters of 15 µm to 20 µm) and the temporal scale to 1 MCS ≈ 3.1 min, we achieved simulated leader velocities of approximately 0.32 µm/min at λ=20 and 0.9 µm/min to 1.2 µm/min at λ≈30, matching experimental measurements. Our standard simulation duration of 700 MCS represents approximately 36 hours of biological time, consistent with SaGA-based invasion imaging [12,18].

Volume constraints were imposed on all cells with a target volume *V*_0_ = 10 lattice units and volume constraint strength $\lambda_{V}=2.0$. The volume constraint strength was chosen to allow realistic cell shape fluctuations while preventing unrealistic cell fragmentation or excessive volume deviation, following previous CPM tumor models [36]. The main simulation study examined the impact of three biophysical control parameters on tumor invasion dynamics. The first parameter, leader–follower contact energy ($J_{lf}$), regulated the adhesive interaction strength between LCs and FCs and was varied across a range of −5 to +5 in 11 discrete levels. In the CPM, higher contact energy ($J_{lf}$) corresponds to weaker adhesion between cell types, while lower (more negative) $J_{lf}$ indicates stronger adhesive interactions. The second parameter, the migration coefficient (λ), modulated the response of LCs to the chemoattractant gradient, ∇$c(\vec{x})$. We varied λ from 0 to 30 in 11 discrete levels, it represents the migratory sensitivity and can be interpreted as the effective force per unit concentration gradient experienced by leader cells. The third parameter, proliferative probability (*PP*), represented the fraction of FCs that are proliferation-competent, with values ranging from 0 to 1.0 in 11 discrete levels.

These parameter ranges were identified through preliminary exploration spanning the full spectrum of qualitatively distinct invasion phenotypes. Preliminary simulations (*n* = 50 across broader ranges: $J_{lf}\in [-10,10]$, λ∈[0,50], PP∈[0,1]) revealed that parameter values outside the selected ranges produced either uniform behavior or computational artifacts. Specifically, $J_{lf}<-5$ resulted in uniformly static, non-invasive tumors due to excessive cell–cell cohesion, while $J_{lf}>5$ caused complete tumor fragmentation into isolated cells. Similarly, λ>30 induced boundary artifacts due to excessively rapid cell displacement, exceeding the lattice resolution. At the lower end, tumors remained compact with minimal morphological variation. The refined ranges ($J_{lf}\in [-5,5]$, λ∈[0,30]) were chosen to span regimes capable of producing all four invasion modes (no invasion, single-cell, bulk collective, multimodal).

Each of the 11^3^ = 1331 unique parameter combinations was replicated 10 times using unique random seeds (0–9), yielding 13 310 total simulations. Follower cell proliferation was implemented using a clock-based system: at the start of the simulation, a subset of size $PP\times N_{FC}$ (where $N_{FC}\approx 1200$ is the initial follower count) was designated as proliferation-competent. Proliferation-competent cells underwent mitosis when their volume exceeded 20 pixels (twice the target volume) and their internal clock surpassed a division time drawn uniformly from 75 ± 50 MCS, i.e., 𝒰(25,125) MCS,corresponding to a mean inter-division time of 75 MCS≈3.1 h. This accelerated proliferation rate reflects rapid growth in aggressive cancer cell lines cultured in 3D ECM [43]. Upon division, cells were bisected along random axes, and daughter cells inherited a proliferation-competent state with reset timers. Leader cells were not assigned proliferation capacity, consistent with experimental observations that highly motile, invasive leader cells often exhibit reduced proliferation compared to followers [12]. Parameter values were motivated by experimental estimates from biological observations on collective cancer invasion [3,12]. A complete summary of all simulation parameters, including dimensions, fixed contact energies, and control parameters, is provided in Table 1.

**Table 1:** Simulation and biophysical parameters used in the leader–follower tumor invasion model. Dimensions are expressed in terms of *L* (length), *T* (time), and *E* (energy). Parameters marked with an asterisk (^*^) were systematically varied during the parametric scan. Superscript notation indicates: (i) calibrated to experimental data, (ii) selected from preliminary parametric exploration, (iii) based on literature values or experimental observations, (iv) standard CPM framework parameters.

Parameter	Symbol	Model Value	Dimension
Boltzmann temperature	*T*	10.0^(iv)^	*E*
Leader cell fraction	*k*	25%^(iii)^, 15%, 35%	—
Follower growth rate	*f*_grow_	0.015 MCS^−1 (iii)^	*L*^2^/*T*
Lattice size	—	500×300	*L* ^2^
Volume constraint strength	$\lambda_{V}$	2.0^(iv)^	*E*/*L*^2^
Target cell volume	*V*_0_	10 px^(iii)^	*L* ^2^
Migration strength^*^ (leaders)	λ	[0, 30]^(i,ii)^	*E*/*L*
Proliferative probability^*^	*PP*	[0, 1.0]^(iii)^	—
Contact energy^*^: Leader–Follower	$J_{lf}$	[−5, + 5]^(ii)^	*E*
Contact energy: Medium–Leader	$J_{ml}$	2.0^(iv)^	*E*
Contact energy: Leader–Leader	$J_{ll}$	16.0^(iv)^	*E*
Contact energy: Follower–Follower	$J_{ff}$	5.0^(iv)^	*E*
Contact energy: Medium–Follower	$J_{mf}$	10.0^(iv)^	*E*

### Computational implementation and code availability

Post-processing used NumPy 1.21, SciPy 1.7, NetworkX 2.6, and Matplotlib 3.4. Each simulation ran for 700 MCS with periodic boundary conditions along the x-axis, requiring approximately 12–18 minutes per replicate on an Intel Xeon E5-2680 processor (2.4 GHz, 8 cores). The complete parameter sweep required approximately 2,800 CPU-hours, distributed across a 48-core computing cluster. Simulations were executed in batches using environment variables to specify parameter combinations (J_LF, MU, PP) and replicate indices (REP), enabling efficient parallelization. All simulation code, post-processing scripts, and analysis notebooks are available in the GitHub repository: https://github.com/Jiang-Lab/Tumor_Invasion_Model.

### Invasion metrics and phenotype classification

For each simulation, spatial and topological features were extracted at the final time point (MCS = 700) using a combination of breadth-first search (BFS), neighborhood analysis, morphological processing, and cell-type–specific annotation. The main tumor mass was identified by constructing a contact graph in which each cell is represented as a node and cell–cell contacts define edges. Starting from all cells at the domain base (*y* = 1), we performed a BFS to identify all physically connected cells, thereby defining the set of main tumor cells. Cells were considered neighbors if they shared at least one lattice edge (von Neumann neighborhood). This graph-based approach naturally captures the connected component corresponding to the primary tumor while excluding spatially detached clusters and solitary defectors.

**Fig 2:**
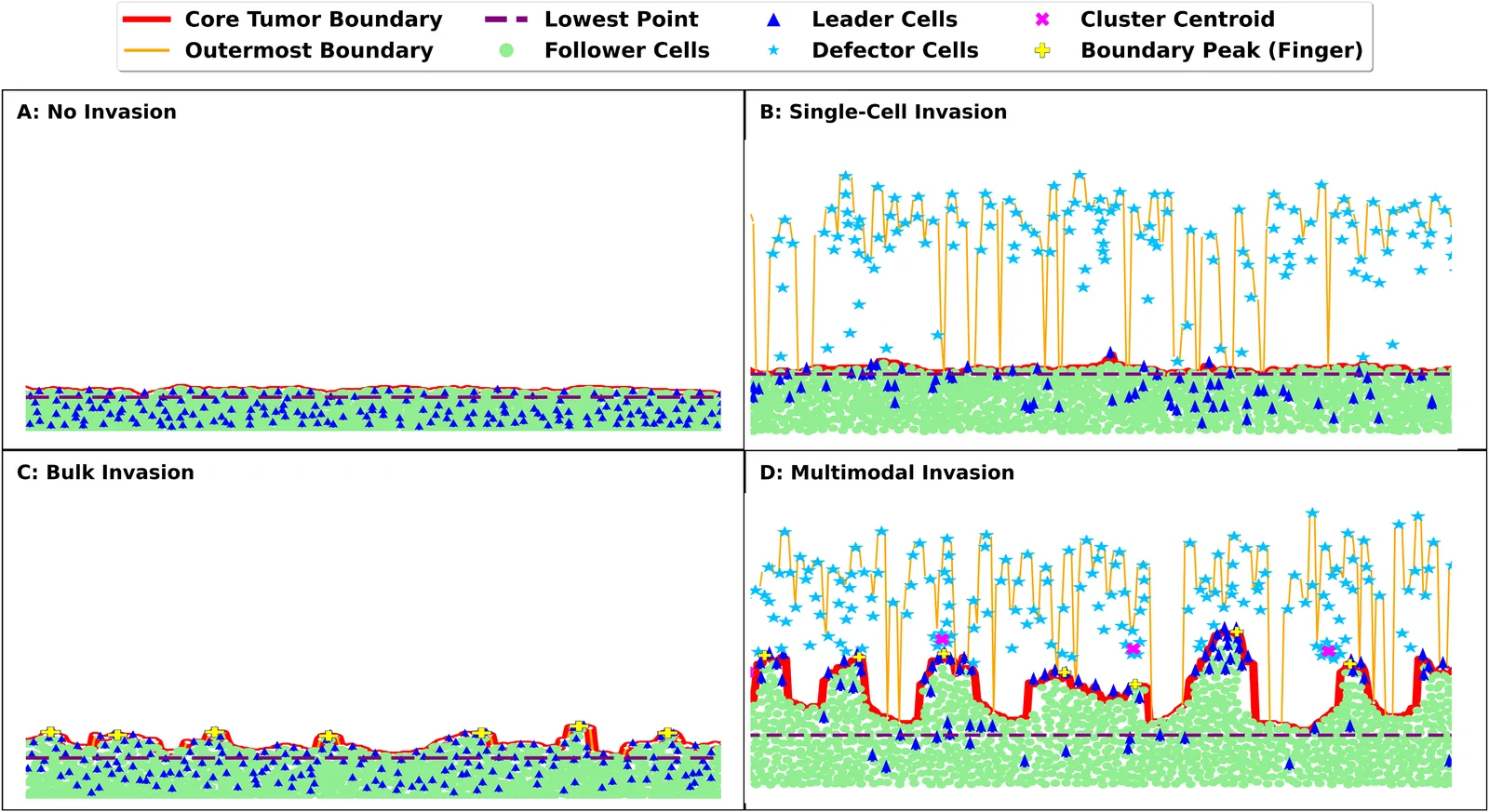
Distinct emergent invasion phenotypes in leader–follower tumor simulations. Representative snapshots of tumor morphologies at endpoint (MCS = 700) for the four canonical invasion phenotypes, each arising from unique combinations of leader motility, follower proliferation, and intercellular adhesion parameters. **(A)** No Invasion: Tumor remains compact with no detachment or dispersal beyond initial bounds. Invasive and infiltrative areas are nearly identical; fingers, defectors, and clusters are absent. **(B)** Single-Cell Invasion: Individual cells (cyan stars) detach from the tumor mass, increasing the infiltrative area without forming collective structures. **(C)** Bulk Invasion: Finger-like protrusions (yellow pentagons with navy edges) emerge, indicative of coordinated collective migration; defectors and clusters are not observed. The invasive area is large and contiguous. Fingers are defined as protrusions ≥10 pixels (~30 µm) in the y-direction, separation of at least 20 pixels (~ 60 µm) from neighboring peaks in the x-direction, and width at half-height prominence ≥5 pixels. **(D)** Multimodal Invasion: The coexistence of collective fingers, solitary defectors, and detached clusters (magenta Xs) reflects a heterogeneous expansion mode that combines cohesive and dispersed strategies. Standard annotations across panels: core boundary (red), outermost boundary (orange), lowest point (purple dashed), follower cells (light green circles), leader cells (blue arrows), defector cells (cyan stars), cluster centroids (magenta **X)**, boundary peaks (yellow pentagons with navy edges).

The **invasive area** was defined as the cumulative area occupied by the main tumor mass [18], computed by summing the volumes of all cells belonging to the connected component anchored at *y* = 1. This metric reflects cohesive tumor expansion and requires cells to maintain physical contact with the core structure. The invasive front boundary is shown by the red curve in Fig 2. The **infiltrative area** was computed as the area of the convex hull enclosing all tumor cells (both main tumor and detached), capturing the overall spatial footprint of the tumor, including dispersed single cells and clusters [44]. This metric provides a global measure of spatial dispersion, irrespective of physical connectivity, and is represented by the orange outermost boundary in Fig 2.

To characterize morphological complexity, we extracted geometric features from the simulated tumor front. For each x-coordinate, we scanned vertically from the top of the domain (*y* = 299) downward to identify the highest y-position occupied by a main tumor cell, yielding the tumor boundary profile *y*_tumor_(*x*). This one-dimensional boundary was interpolated using cubic smoothing splines (SciPy) to facilitate derivative calculations and peak detection. **Invasive fingers** were detected using a peak-finding algorithm applied to the interpolated boundary profile. Peaks were classified as fingers if they satisfied three criteria; prominence ≥10 pixels in the y-direction (corresponding to ~30 µm) protrusion height), separation of at least 20 pixels (~ 60 µm) from neighboring peaks in the x-direction, and width at half-prominence ≥5 pixels. These thresholds were chosen to identify biologically meaningful finger-like protrusions while filtering spurious peaks arising from single-cell fluctuations, following established definitions in prior computational studies [45,46]. To further reduce over-counting of closely spaced peaks, we applied a post-processing merge step: peaks separated by less than 15 pixels were consolidated into a single finger, retaining only the leftmost peak. The resulting count of merged peaks constituted the final finger metric.

**Singles** were defined as individual cells (leader or follower) with no contact neighbors. Specifically, a cell was classified as a single defector if it shared no lattice edges with any other tumor cell (von Neumann neighborhood criterion) and was located above the minimum y-coordinate of the main tumor. This definition captures solitary cells that have fully detached and migrated away from any collective structure. **Detached clusters** were operationally defined as spatially separated groups of two or more tumor cells that maintained internal connectivity but were entirely disconnected from the main tumor component [47]. Clusters were identified using BFS on all cells not belonging to the main tumor: for each follower cell outside the main tumor, we initiated a BFS to identify its connected component. If this component contained two or more cells and had no contact with the main tumor, it was classified as a detached cluster. Cluster composition was quantified by counting the number of leaders and followers within each cluster, enabling classification of purely follower-based aggregates, leader-only satellites, and mixed clusters. Cluster centroids were computed as the mean x- and y-coordinates of member cells. Cluster persistence time was measured from emergence to either dissolution (loss of all intercellular connections within the cluster or complete reintegration into the main tumor mass) or right-censoring at the simulation endpoint (*t* = 700 MCS). Clusters persisting until the final time point were recorded separately to distinguish early-forming clusters with complete dissolution histories from late-forming clusters constrained by the finite simulation window. The majority of the clusters lived through the end of the simulation; therefore, longer persistence time corresponds to earlier cluster formation.

Representative examples of the annotated morphological features associated with each invasion phenotype are shown in Fig 2. Each simulation was assigned a phenotypic label using a rule-based classification framework designed to identify biologically interpretable modes of tumor invasion. The classification used endpoint invasion metrics measured at MCS = 700, including invasive area, infiltrative area, finger-like protrusions, solitary defectors, and detached clusters. Importantly, invasion was defined operationally by a morphological transition from a smooth, compact tumor boundary to a boundary displaying protrusions, cellular detachment, or dispersed infiltrative structures, rather than by a fixed distance threshold from the tumor core. Using these criteria, simulations were categorized into four phenotypes: No Invasion, Single-Cell Invasion, Bulk Invasion, and Multimodal Invasion. This scheme was designed to parallel experimental classifications of tumor invasion behavior [5].

Simulations were classified as No Invasion (Fig 2A) when the invasive and infiltrative areas were nearly identical, and no fingers, solitary defectors, or detached clusters were detected, indicating that the tumor remained morphologically compact and did not substantially breach its initial boundary. The Single-Cell Invasion phenotype (Fig 2B) was assigned when the infiltrative area exceeded the invasive area and solitary defectors were present, while finger-like protrusions and detached clusters were absent. This phenotype represents dispersal of individual cells without coordinated collective invasion. Simulations were classified as Bulk Invasion (Fig 2C) when they exhibited a large invasive area with finger-like protrusions but no solitary defectors or detached clusters, indicating coherent, strand-like collective expansion of the tumor front. Finally, the Multimodal Invasion phenotype (Fig 2D) was assigned when cohesive invasive structures, such as fingers, coexisted with dispersed elements, such as solitary defectors or detached clusters, and the infiltrative area substantially exceeded the invasive area. This phenotype captures a hybrid invasion mode in which collective and individual invasion occur within the same spatial domain.

This automated classification pipeline enabled the high-throughput categorization of invasion phenotypes across the whole parameter space of 13 310 simulations. We saved the outputs in CSV format and compiled them into aggregate data tables for phenotype classification. The robustness of classifier thresholds was confirmed by varying finger-prominence and invasive-to-infiltrative area ratio criteria by ±20% from baseline values; phenotype boundaries and classifications remained stable across the parameter space.

## Results

Using this calibrated leader–follower CPM framework, we performed a high-throughput sweep over adhesion, motility, and proliferation to map invasion phenotypes and quantify dissemination metrics.

### Distinct invasion metric sensitivities to adhesion, motility, and proliferation

To dissect how cellular biophysical properties shape emergent tumor invasion dynamics, we systematically varied leader–follower contact energy ($J_{lf}$), leader migration coefficient (λ), and follower proliferative probability (PP) across a three-dimensional parameter space encompassing 13 310 simulations (1,331 unique parameter combinations with *n* = 10 replicates each). Each simulation ran for 700 MCS (approximately 36 h of biological time), and we quantified five invasion metrics at endpoint: invasive area (expansion of the connected tumor mass), infiltrative area (total spatial footprint including dispersed cells), number of finger-like protrusions, single invading cells (singles), and detached clusters. These metrics capture distinct aspects of tumor spatial organization and collectively define invasion phenotypes.

Invasive area, measuring the expansion of the connected tumor mass, was maximized under intermediate adhesion ($J_{lf}\approx 0$ to 2) and elevated migration (λ≥15) (S2 Fig). Strong adhesion significantly reduced invasive expansion compared to intermediate adhesion. Under strong adhesion conditions ($J_{lf}\leq -2$), mean invasive area was 33 875 µm^2^, representing a 1.9-fold reduction compared to intermediate adhesion which yielded 64 463 µm^2^. Weak adhesion ($J_{lf}>2$) further reduced invasive area to 41 856 µm^2^, which is 1.5-fold lower than intermediate adhesion. Migration exhibited a substantially stronger effect on invasive expansion. High migration (λ≥20) produced a 4.2-fold increase in invasive area compared to low migration (λ<10). Specifically, high migration conditions generated mean invasive area of 76 125 µm^2^ whereas low migration yielded only 18 163 µm^2^. Strong adhesion prevented structural remodeling, while very weak adhesion reduced cohesion, both of which limited expansion. Higher PP increases follower cell mass within the tumor, whereas adhesion and leader motility primarily determine invasion mode.

In contrast, the infiltrative area, which captures the full spatial footprint, including dispersed single cells and clusters, was dictated primarily by migration. The Infiltrative area increased monotonically with the migration coefficient and exhibited an 8.5-fold increase from low to high migration conditions. Low migration (λ<10) produced mean infiltrative area of 22 369 µm^2^ while high migration (λ≥20) generated 191 200 µm^2^(S3 Fig). Adhesion also modulated infiltrative spread under high migration conditions. Weak adhesion ($J_{lf}>2$) increased infiltrative area to 183 406 µm^2^, representing a 1.7-fold increase compared to intermediate adhesion. Thus, directional motility alone is sufficient for extensive tumor spread, although it does not necessarily lead to cohesive growth.

Finger counts peaked at intermediate adhesion ($J_{lf}\approx 0$ to 2), yielding a marginal mean of 7.11 fingers (averaged across all migration coefficients and proliferative probabilities), and at strong migration (λ≥20) with a marginal mean of 9.74 fingers (averaged across all adhesion strengths and proliferative probabilities) (S5 Fig). Strong adhesion ($J_{lf}\leq -2$) restricted protrusive branching (mean = 4.03 fingers), while weak adhesion ($J_{lf}>2$) fragmented collectives into singles or clusters (mean = 6.11 fingers). This non-monotonic dependence on adhesion is consistent with experimental studies showing that optimal intercellular adhesion promotes dynamic collective invasion [45,46]. Proliferation had a negligible influence on finger formation (Pearson r=−0.000, *p* = 0.985). The number of singles increased sharply with λ and weak adhesion ($J_{lf}>2$), reflecting a transition toward mesenchymal-like escape (S4 Fig). Weak adhesion produced mean = 175.79 singles compared to 1.53 under strong adhesion and 53.06 under intermediate adhesion. While such single cells enable early dissemination, clinical evidence suggests that solitary circulating tumor cells frequently enter dormancy or undergo apoptosis, thereby limiting their metastatic potential [48].

Detached clusters, defined as cohesive aggregates that separate from the core, formed in 28.3% of all simulations (3 763 out of 13 310) and were most prevalent under intermediate-to-weak adhesion ($J_{lf}=0$ to 3) and high migration (λ≥24) (S6 Fig). Intermediate adhesion produced a mean of 1.69 clusters per simulation, while weak adhesion yielded 1.78 clusters. High migration (λ≥24) resulted in 70.1% of simulations exhibiting clusters, with mean of 5.00 clusters when present. In this regime, adhesion is strong enough to preserve intercellular cohesion within the aggregate yet weak enough to allow detachment from the primary mass. Larger clusters tended to persist until the end of simulations (700 MCS), while smaller aggregates often dissolved earlier. Again, proliferation (PP) showed no consistent effect on cluster frequency or stability (Pearson r=−0.002, *p* = 0.815). These observations parallel experimental findings that cluster-based dissemination is highly metastatic [47,49].

Correlation-based sensitivity analysis (S7 Fig) corroborated these trends: migration showed the strongest correlations with invasive area (Pearson *r* = 0.70, Spearman ρ=0.80), infiltrative area (Pearson *r* = 0.69, Spearman ρ=0.80), and finger counts (Pearson *r* = 0.80, Spearman ρ=0.81). Contact energy exhibited moderate-to-strong correlations with singles (Pearson *r* = 0.67, Spearman ρ=0.71) and infiltrative area (Pearson *r* = 0.57, Spearman ρ=0.50). Proliferation exhibited weak correlation across all metrics (Pearson and Spearman |*r*| < 0.03, all *p* > 0.05), reinforcing its limited role in determining invasion mode. These metric sensitivities were qualitatively preserved across simulations run at leader fractions of 15% and 35%, confirming that the reported parameter dependencies are not specific to the 25% baseline condition (S9 Fig).

**Fig 3:**
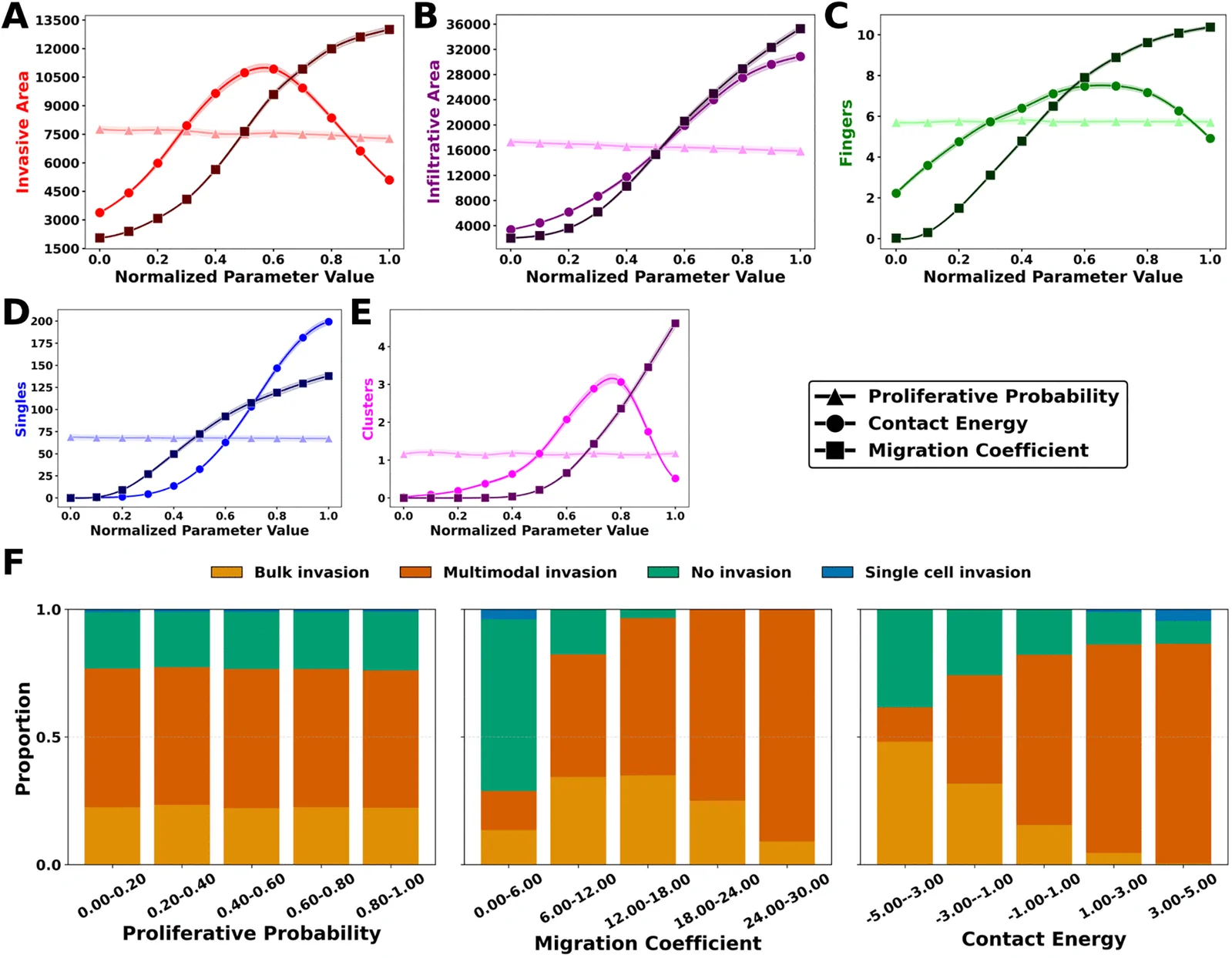
Migration and adhesion together determine invasion modes, while proliferation scales tumor bulk. **(A–E)** Metric parameter relationships for invasive area **(A)**, infiltrative area **(B)**, fingers **(C)**, singles **(D)**, and detached clusters **(E)**. The x-axis is a normalized parameter value (scaled to [0,1]). Original parameter value ranges were λ∈[0,30] (migration coefficient) and $J_{lf}\in [-5,5]$ (contact energy, lower values = stronger adhesion). Curves show the marginal dependence on proliferative probability(triangles, ▴), contact energy (circles, •), and migration coefficient (squares, ∎), all plotted as solid lines. Shaded bands denote mean ± standard error of the mean (SEM). Migration strongly increased invasive and infiltrative areas, singles, and clusters. Contact energy shows non-monotonic optima at intermediate adhesion, and proliferation induces minor changes. **(F)** Phenotype composition across proliferative probability (PP) bins, computed as normalized frequency counts across all 13,310 simulations, with error bars denoting mean ± s.d. across ten replicates per parameter combination. Phenotype proportions remain largely unchanged across PP bins, whereas migration (λ) and adhesion ($J_{lf}$) drive substantial phenotype transitions.

To directly examine the marginal effects of proliferative probability on invasion metrics, we systematically compared metric-parameter relationships across all three parameters (Fig 3A-3E). Across all five invasion metrics, PP showed only minimal influence compared with the migration coefficient and contact energy. Increasing the migration coefficient (λ) consistently elevated the invasive and infiltrative area, promoted the formation of fingers, and sharply increased the number of singles and detached clusters. Contact energy ($J_{lf}$) exerted a non-monotonic effect, with peaks at intermediate adhesion ($J_{lf}\approx 0$–2) that supported cluster formation and cohesive protrusions. By contrast, proliferative probability produced nearly flat curves, with only minor oscillatory deviations. While high proliferation increases follower availability and may create local crowding effects in the tumor core, the overall prevalence of invasion phenotypes and cluster frequency show no systematic dependence on PP. This suggests that mechanical parameters (adhesion and migration) are the primary determinants of invasion architecture. Thus, proliferation increased overall tumor bulk but did not enhance the spatial extension of the connected invasion front.

To validate that these metrics capture consistent invasion features across geometries, we compared representative parameter combinations from each phenotype in 2D and 3D simulations: no invasion ($J_{lf}=-2,\lambda =5,PP=0.5$), single-cell invasion ($J_{lf}=4,\lambda =10,PP=0.5$), bulk invasion ($J_{lf}=-2,\lambda =15,PP=0.5$), and multimodal invasion ($J_{lf}=2,\lambda =30,PP=0.5$). Invasive area, finger counts, and cluster incidence differed by <15% between geometries across all phenotypes, confirming that the 2D domain preserves key invasion features across the parameter space explored. Together, these findings demonstrate leader cell motility and intercellular adhesion as the primary regulators of invasion architecture, with proliferation acting mainly as a scaling factor for tumor bulk. This functional decoupling underscores the central role of mechanical and migratory properties in shaping invasion phenotype and spatial spread.

### Phenotypic phase-space mapping reveals prevalence of multimodal invasion

We classify emergent invasion behaviors within our leader-follower framework into four biologically interpretable phenotypes: No Invasion, Single-Cell Invasion, Bulk Invasion, and Multimodal Invasion. Phenotype labels were assigned by a rule-based classifier applied to multiple invasion metrics, including invasive area, infiltrative spread, finger counts, single cells, and detached clusters. The classification thresholds were grounded in biological principles: the infiltrative-to-invasive area ratio distinguishes phenotypes by definition; when these areas are nearly equal, no cellular dispersion occurs (no invasion or bulk invasion); when the infiltrative area substantially exceeds the invasive area, cells have dispersed as single cells or in a multimodal mix. Finger-prominence thresholds (≥10 pixels height, ≥20 pixels separation) follow established definitions [45,46]. To assess robustness, we spot-checked representative parameter combinations from each phenotype and varied thresholds by ±20% (e.g., finger height 8−12 pixels). Phenotype classifications remained stable because phenotypes are defined by combining multiple criteria simultaneously, making them insensitive to minor adjustments in any single metric. This approach provides an objective mapping of complex invasion behaviors across the parameter landscape.

**Fig 4:**
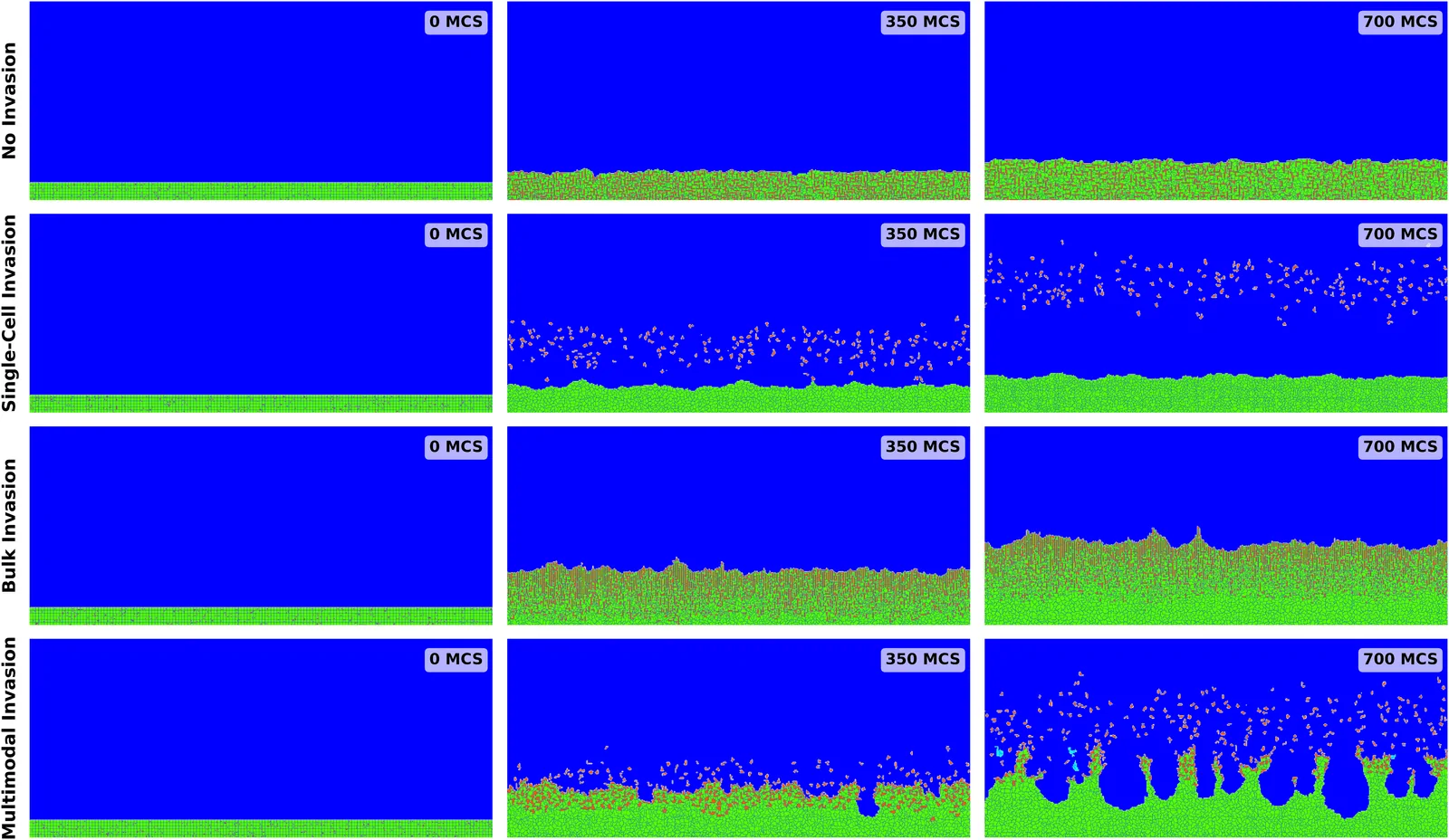
Temporal evolution of tumor morphologies across invasion phenotypes. Rows show representative simulations for the four phenotypes, no invasion, single-cell invasion, bulk invasion, and multimodal invasion, while columns show time points (0, 350, and 700 MCS, corresponding to 0, 18, and 36 hours). Leader cells (red) respond to the vertical gradient, and follower cells (green) proliferate and maintain cohesion. In multimodal invasion, cyan represents detached clusters. **No invasion** (top row) remains compact, illustrating regimes of strong adhesion and negligible leader motility. **Single-cell invasion** (second row) emerges under weak adhesion and high leader motility, where leader cells detach and migrate individually. **Bulk invasion** (third row) features cohesive, finger-like protrusions that preserve junctional integrity, supported by high motility and proliferative replenishment. **Multimodal invasion** (bottom row) exhibits coexisting fingers, dispersed singles, and detached clusters, under intermediate adhesion and high leader motility. See S2 Video for full spatiotemporal dynamics.

These categories align with the framework proposed by Friedl *et al.* [5], which organizes invasion behavior along a spectrum from single-cell migration to collective streaming. Our Single-Cell Invasion corresponds to single-cell escape, in which leader cells detach and migrate independently from the core tumor mass under weak adhesion. Bulk Invasion reflects collective streaming behavior, characterized by coordinated protrusions and preserved junctional integrity. No Invasion represents non-migratory, growth-dominant behavior. Finally, Multimodal Invasion shows the richest dynamics, with the simultaneous formation of collective fingers, single cells, and detached clusters, a phenotype increasingly observed in aggressive clinical tumors [49,50] but rarely captured by computational models. While our model captures these principal invasion modes through the mechanical balance of adhesion, motility, and proliferation, it represents a focused subset of the broader Friedl framework, abstracting away from features such as morphological plasticity, explicit multicellular polarity, and dynamic ECM remodeling that would require additional mechanistic complexity. Figs 4 and S8 illustrate how these phenotypes emerge over time.

**Fig 5:**
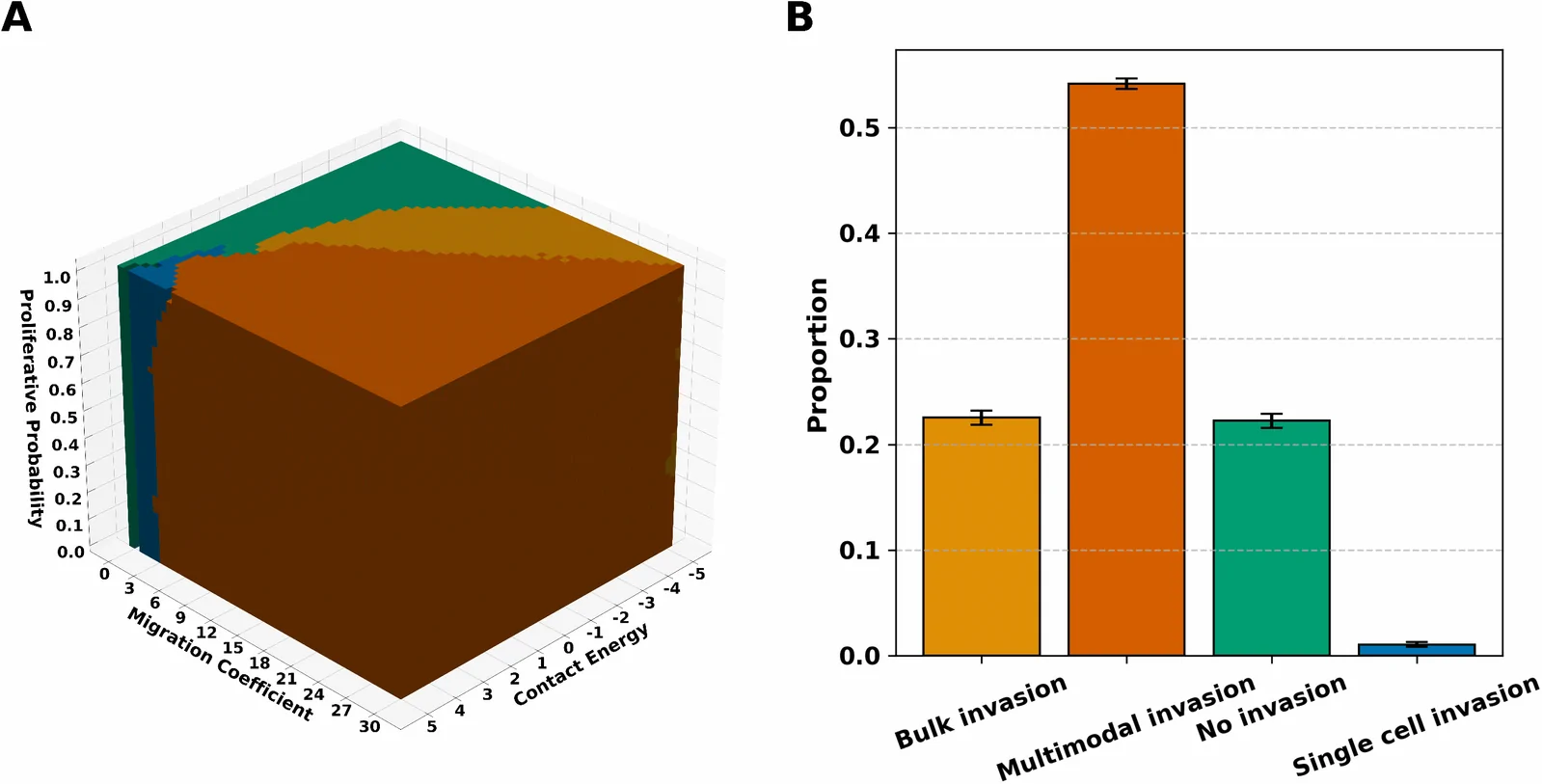
3D phase space of invasion phenotypes. (**A**) Multidimensional phenotype map over leader–follower contact energy ($J_{lf}$), leader migration strength (λ), and follower proliferative probability (PP). Each voxel is assigned the most probable phenotype: green (No Invasion), blue (Single-Cell Invasion), orange (Bulk Invasion), or gold (Multimodal Invasion). Boundaries between regimes are sharp and nonlinear, and multimodal invasion occupies a large region characterized by moderate adhesion and strong migration across all PP. See S8 Fig for 2D cross-sections of the phase space. (**B**) The phenotype frequency distribution across 13 310 simulations. Multimodal invasion is the most prevalent outcome, with bulk and no invasion occurring under more restricted conditions, and single-cell invasion is comparatively rare. Error bars denote mean ± s.d. across 10 replicates. S3 Video shows the temporal progression, with early compact states giving way to increasing multimodal prevalence at later times.

We visualized phenotype assignments in the 3D parameter space defined by leader–follower contact energy ($J_{lf}$), leader migratory coefficient (λ), and follower proliferative probability (PP). The resulting phase map (Fig 5A) reveals well-delineated phenotypic regions separated by sharp, nonlinear transitions as one or more control parameters vary. As summarized in S1 Table, the No Invasion phenotype (green) occupies approximately 22% of the parameter space primarily at low migration (λ<10) and moderate to strong adhesion ($J_{lf}\leq 2$), where leader cells either lack migratory drive or remain tightly bound to follower cells, preventing outward expansion. Single-Cell Invasion (blue) appears in a narrow but distinct band (~1% of parameter space) at weak adhesion ($J_{lf}>3$) and low to intermediate migration (4≤λ≤12), where leaders detach and migrate independently with limited follower engagement. Bulk Collective Invasion (orange) emerges in approximately 23% of parameter space under strong adhesion ($J_{lf}\ll 0$), moderate migration (λ≈10−20), and moderate proliferation (0.1 < *PP* < 0.7), producing cohesive strands resembling the leader-follower chains observed in NSCLC spheroids [12]. In contrast, Multimodal Invasion (gold) spans a broad range (~54% of parameter space) of intermediate to weak adhesion ($-1\leq J_{lf}\leq 5$), high migration (λ>15), and moderate to high proliferation (*PP* > 0.3), representing the most prevalent invasion outcome in our simulation.

Notably, multimodal invasion is the most prevalent outcome in our simulations (Fig 5B), with more than half of all parameter combinations favoring this heterogeneous phenotype. This suggests that heterogeneous invasion, characterized by coexisting finger-like protrusions, detached clusters, and isolated individual cells, is a robust and recurrent tumor behavior. This phenotype emerges most frequently under intermediate adhesion and high motility, consistent with histopathological and experimental observations in invasive carcinomas, where collective strands often coexist with solitary migratory cells and multicellular clusters [10,49]. While specific tumor subtypes may differ in their parameter mappings, the phase structure suggests testable strategies for shifting tumors away from multimodal regions by jointly modulating adhesion and motility.

To examine the phenotypic impact of proliferation more directly, we assessed phenotype classification across the full parameter space. Across all simulations (13,310 total), the proportion of tumors in the bulk, multimodal, single-cell, and non-invasive classes remained essentially unchanged across proliferative probability (PP) bins. In sharp contrast, both λ and $J_{lf}$ produced marked phenotype transitions. Statistical sensitivity analyses reinforced these observations: distance correlation analysis revealed strong dependencies of invasion metrics on migration (up to 0.79 for fingers) and moderate dependencies on adhesion (e.g., 0.68 for singles), while PP correlations were negligible (≤0.02) (S7 Fig). A multi-output random forest model trained on all three parameters (400 trees, mean-decrease-in-impurity criterion) showed a similar pattern: migration (~0.51) and adhesion (~0.49) accounted for virtually all explanatory power, while proliferation contributed negligibly (~0.01). Pearson and Spearman correlation coefficients similarly showed near-zero correlations between PP and all metrics (|*r*| < 0.03). Full statistical analysis methods, including distance correlation, random forest importance, and SEM computation, are provided in S1 Appendix. These results demonstrate that while proliferation affects the scale of tumor growth (reflected in a larger invasive area at high PP due to core expansion), it does not alter the phenotypic classification or the fundamental structure of invasion modes. Migration and adhesion remain the primary regulators of spatial architecture and invasion phenotype.

### Cluster formation peaks under intermediate adhesion and strong migration

Multicellular clusters represent a distinct invasion mode capable of cohesive dissemination from the primary tumor mass. Clusters typically preserve leader–follower heterogeneity, wherein leader cells provide traction and directional persistence while followers maintain cohesion. Understanding the mechanical principles governing cluster formation in vitro provides a foundation for investigating multicellular escape strategies in more complex environments.

**Fig 6:**
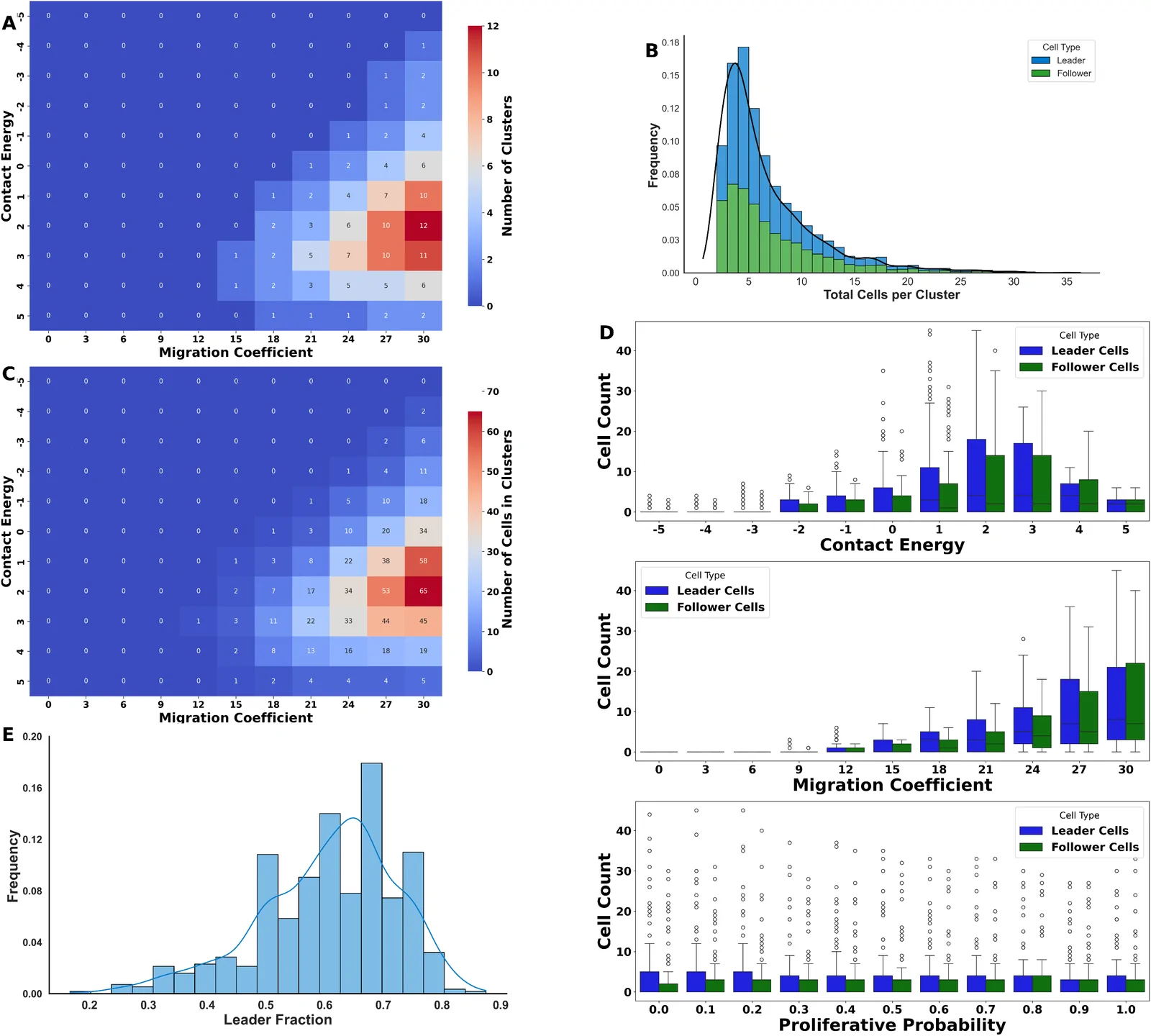
Cluster formation landscape: incidence, abundance, and composition across parameter space. **(A)** Heatmap of cluster incidence (number of clusters per simulation) across adhesion ($J_{lf}$, y-axis) and migration (λ, x-axis). Each cell represents the mean count across 10 replicates. Blue indicates no or few clusters; red indicates high cluster frequency, peaking at intermediate adhesion ($J_{lf}\approx 0$–2) and high migration (λ≥24). **(B)** Histogram of cells per cluster showing the distribution of cluster sizes. Most clusters contained 4–8 cells, with rare aggregates exceeding 30 cells, with an average of ~7 cells. **(C)** Heatmap of total cells per cluster (mean per simulation) showing that higher cluster incidence is associated with large aggregate sizes. Peak values (30–40 cells/cluster) occur in the same intermediate-adhesion, high-migration window. **(D, E)** Cluster composition analysis showing leader and follower cell counts across parameter space.

To examine the mechanistic drivers of cluster detachment, we systematically evaluated simulation outcomes and computed cluster-related metrics at every 10 MCS, including cluster counts, size distributions, cellular composition, and trajectory persistence, across 13 310 simulations. Cluster emergence depended strongly on the adhesion-motility balance. Heatmaps of cluster incidence (Fig 6A) showed a sharp increase under intermediate adhesion ($J_{lf}\approx 0$–3) and strong motility (λ≈24–30). The heatmap of total cells aggregated in clusters (Fig 6C) further revealed that this regime not only produces more cluster-forming events but also results in larger clusters: high-migration, intermediate-adhesion conditions yielded 30–65 cells in clusters on average, compared to fewer than 5 cells in low-migration or very-weak-adhesion regimes. These conditions define a mechanically permissive regime in which adhesion is low enough to permit detachment yet high enough to preserve intra-cluster cohesion, while high leader motility provides leader-driven traction for escape. By contrast, strong adhesion ($J_{lf}<-2$) or low motility (λ<15) suppressed cluster formation, confining cells to the primary tumor mass.

Cluster composition showed substantial leader enrichment. Analysis across all parameter combinations revealed that leader fractions commonly reached approximately 60–70% in the cluster-forming regime, suggesting that leaders were consistently enriched within clusters, with a median of approximately 4 leaders per cluster versus 3 followers (Fig 6B). The leader fraction distribution (S13 Fig) exhibited consistent enrichment across proliferative probability values, peaking at approximately 70% in the multimodal invasion regime, indicating that clusters are not random aggregates but leader-dominated collectives with robust composition. This enrichment reflects the mechanistic role of leader cells in generating traction and directional persistence.

Across the explored range, PP exerted at most a secondary influence on cluster incidence. Varying PP altered the follower availability but did not qualitatively change the adhesion–motility trends or cluster stability. Fixing *PP* = 0.9 in parameter scans confirmed that proliferation primarily increased follower counts within clusters without eliminating leader dominance. This finding suggests that motility and adhesion, rather than proliferation, are the principal determinants of cluster detachment.

**Fig 7:**
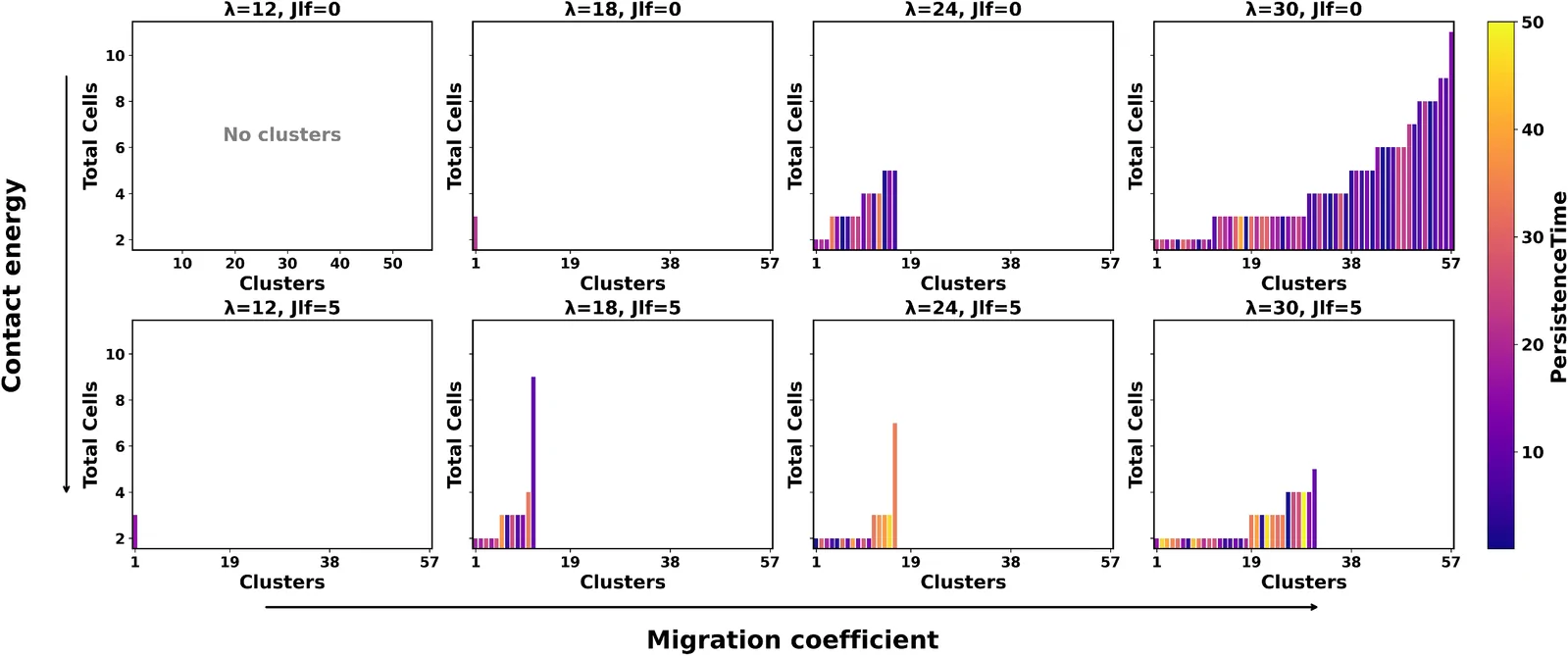
Representative cluster size distributions across migration and adhesion at fixed proliferation. Shown for *PP* = 0.9 at a subset of $\left(\lambda ,J_{lf}\right)$ combinations chosen to illustrate regimes with and without cluster formation. (See S11 Fig for the full panel.) Each panel shows the distribution of cluster sizes (y-axis) versus the number of clusters observed (x-axis), pooled across *n* = 10 replicates and sampled every 10 MCS. Bars are colored by persistence time, with lighter shades indicating longer-lived clusters. Persistence time is expressed in sampling units of 10 MCS, such that a value of 50 corresponds to 500 MCS of biological simulation time. Panels are arranged in the $\left(\lambda ,J_{lf}\right)$ plane, with migration coefficient increasing from left to right and contact energy increasing from top to bottom, as indicated by the outer axis arrows. Panels labeled “No Clusters” indicate conditions under which no clusters formed. Across the representative conditions, strong-adhesion or low-motility regimes show few or no clusters, whereas intermediate adhesion ($J_{lf}\approx 0$–2) combined with high motility (λ≥24) yields numerous small-to-intermediate clusters and occasional large, persistent aggregates, capturing the heterogeneous outcomes characteristic of this parameter window.

The aggregate distribution of cluster sizes was strongly right-skewed or heavy-tailed (Fig 7). Most clusters consisted of 4–8 cells, while rare aggregates exceeded 30 cells, with a mean cluster size of approximately 7 cells. Persistence time increased with cluster size (S14 and S15 Figs). Most clusters formed within 70 MCS−−140 MCS of the simulation endpoint; because many persisted to 700 MCS, their recorded persistence times are right-censored. Panel insets (S11 Fig) highlight specific parameter combinations (e.g., $\lambda =30,J_{lf}=2$), where small clusters dominated, but rare, large aggregates persisted for the full duration of the simulation, reflecting heterogeneous outcomes characteristic of this regime.

Centroid displacement analyses showed that displacement increased monotonically with the migration coefficient (λ) across the parameter space, with longer-persisting clusters consistently achieving greater total displacement (S12 Fig). At $\left(J_{lf}=1,\;\lambda =30,PP=0.9\right)$, for example, clusters with greater persistence times maintained sustained displacement over time, while shorter-lived clusters remained near their origin (S10 Fig). We additionally tracked splitting events, where one cluster fragmented into smaller aggregates or single cells, and merging events, where detached clusters rejoined another cluster or the tumor margin. Both were most frequent under intermediate adhesion and high motility, where mechanical interactions allowed partial cohesion and dynamic rearrangement, as illustrated in S4 Video. Cluster trajectories, persistence status, and splitting and merging events across all parameter combinations and replicates can be explored interactively via the (Interactive Cluster Trajectory Explorer) https://sheriffacode.github.io/Cluster_Trajectory_Explorer/ described in S2 Appendix.

These results align with experimental observations that collective dissociation is favored in moderately adhesive environments, where leader cells generate traction while followers maintain partial intercellular bonds. The observed enrichment of leader-dominated, intermediate-sized clusters parallels partial epithelial–mesenchymal transition (pEMT), in which cells retain adhesive contacts while acquiring motility. Notably, the cluster size range observed here (mean 4–8 cells, maximum 30–50 cells) is consistent with intermediate-sized multicellular aggregates documented in experimental invasion assays, where such collectives demonstrate enhanced persistence relative to smaller fragments or individual cells. A comprehensive analysis of cluster persistence times across the full parameter space is provided in S11 Fig, showing that the recorded duration is tightly coupled to the same adhesion-motility balance that governs cluster formation. Together, these findings provide a mechanistic explanation for why intermediate adhesion and strong migration create a regime that fosters cluster detachment, leader-driven persistence, and ultimately, metastatic dissemination.

## Discussion

This study identifies several findings that refine the conventional binary distinction between collective and solitary tumor invasion. Across 13,310 simulations, representing 1,331 parameter combinations with ten replicates each, four reproducible phenotypes emerged: No Invasion, Bulk Collective Invasion, Single-Cell Invasion, and Multimodal Invasion. Within the uniformly sampled parameter ranges examined here, Multimodal Invasion occupied the largest region of the phase space (approximately 54%), while purely single-cell invasion was comparatively rare. This prevalence should not be interpreted as an estimate of clinical frequency, because it depends on the parameter ranges and sampling scheme. Rather, it demonstrates that the coexistence of cohesive fingers, detached clusters, and solitary cells is not a fragile or exceptional outcome: it can arise robustly from a small set of mechanical rules. This observation captures a mechanistic subset of the conceptual invasion spectrum proposed by Friedl et al. [5], with patterns whose distinction depends on explicit cell-ECM proteolysis or luminal organization, which are beyond the scope of the current framework.

We highlight four notable findings. First, invasion architecture was controlled primarily by the nonlinear interaction between leader-cell motility and leader-follower adhesion. Migration strongly increased spatial spread, whereas adhesion had a non-monotonic effect: strong cohesion constrained deformation and detachment, very weak cohesion favored cellular dispersal, and intermediate cohesion permitted collective packs to deform, detach, and remain internally connected. Second, follower proliferative probability had little influence on invasion phenotype classification or on the endpoint invasion metrics over the 36-hr simulation window. Third, detached clusters occurred within a mechanically permissive window of intermediate-to-weak adhesion and high leader motility. They were generally enriched for leader cells relative to the 25% initial leader fraction. Fourth, cluster outcomes were strongly heterogeneous: most clusters were small, whereas rare larger aggregates occupied the longer-recorded-duration tail and traveled farther. Together, these findings separate the mechanical control of invasion architecture from the growth-related contribution of follower cells.

The phase diagram provides a mechanistic interpretation of transitions among invasion modes. At low motility, leaders cannot generate sufficient directional traction to reorganize the tumor boundary, and the tumor remains compact. When cohesion is strong and motility is sufficient, leader traction is transmitted through the follower population, producing connected, finger-like protrusions and bulk collective invasion. At the other extreme, weak leader-follower cohesion permits motile cells to detach, increasing single-cell escape and the infiltrative footprint. Multimodal invasion occurs between these limits, where adhesion is weak enough to permit local rupture and detachment but strong enough to preserve multicellular cohesion. The same tumor front can therefore generate fingers, clusters, and solitary cells without requiring separate rules for each behavior. This result explains why intermediate adhesion, rather than maximal or minimal adhesion, produces the largest morphological diversity.

The multimodal phenotype also provides a mechanical complement to partial epithelial–mesenchymal transition (pEMT) [51–54], in which cells co-express epithelial and mesenchymal markers and exhibit a mix of collective and solitary behaviors. Our model does not represent EMT gene-regulatory states; nevertheless, pEMT-like spatial patterns emerge from the effective balance between cohesion and motility alone. This does not imply that transcriptional plasticity is unnecessary in tumors. Instead, it shows that a heterogeneous invasion front can arise even when leader and follower identities are fixed. Coupling the CPM mechanics to gene regulatory models of EMT would allow future studies to determine whether transcriptional plasticity creates new invasion regimes, shifts existing phase boundaries, or stabilizes transition among them.

The analysis of clusters reveals an additional level of organization within multimodal invasion. Cluster composition was not random: in many cluster-forming parameter bins, leaders constituted approximately 60–70% of cluster cells, substantially above the 25% leader fraction in the initial tumor. This enrichment is consistent with preferential recruitment or retention of highly motile cells during detachment, while followers preserve multicellular cohesion. Because some parameter bins contain few clusters and some exhibit leader fractions below 50%, the result is best interpreted as enrichment relative to the starting population rather than universal leader dominance. Cluster duration requires a similarly careful interpretation. Most detected clusters persisted to the simulation endpoint; consequently, their recorded persistence time is right-censored and is strongly influenced by when the cluster first formed. A longer recorded duration therefore often indicates earlier formation rather than a fully observed intrinsic lifetime. The data nevertheless show a robust association between cluster size and the longer-duration tail: small clusters dominate the distribution, whereas rare large aggregates are overrepresented among clusters detected for longer intervals. Longer-recorded-duration clusters also exhibit greater centroid displacement. These observations identify early-forming, larger clusters as a distinct dynamical subset. Additional simulations with longer follow-up or formal survival analysis will be needed to determine whether size or leader composition causally increases cluster stability.

The phase map is most appropriately viewed as a hypothesis-generating framework for controlled experiments. Perturbations that modify E-cadherin/α-catenin-mediated cohesion [55–58] or leader-cell motility pathways such as Rac, Rho/ROCK, and focal adhesion kinase [59,60] could be applied individually and in combination in leader-follower spheroid assays. The model predicts that reducing leader motility should contract the infiltrative footprint, whereas altering cohesion should change the balance among connected fingers, detached clusters, and single cells. These predictions can be tested by quantifying phenotype frequencies, cluster incidence, leader fraction, and cluster trajectories rather than by measuring tumor size alone. Because the current model represents an idealized in vitro system, these proposed interventions should be interpreted as experimental tests of the phase diagram rather than as direct therapeutic recommendations.

Several limitations define the scope of these conclusions and point to natural extensions. First, the 2D slab is a computational simplification of a spheroid invasion assay. A representative 3D simulation reproduces key qualitative structures, including protruding fingers and cluster detachment (S1 Fig, S1 Video), but a systematic parameter sweep in 3D has not yet been performed; quantitative phase boundaries may shift in 3D. Second, the extracellular matrix is static and homogeneous. In reality, cell migration remodels collagen, aligns fibers, and carves guidance tracks that can feed back on motility and cohesion [39,40,61]. Third, leader and follower identities are fixed, follower proliferation is spatially uniform, and the directional cue is prescribed. Phenotypic switching, nutrient limitation, contact inhibition, and competing taxis could generate time-dependent transitions that are absent here. Fourth, endpoint censoring limits inference about cluster lifetime, and longer simulations or time-to-event methods are needed to distinguish early formation from intrinsic persistence. Finally, this model does not include explicit immune or stromal components. Tumor-associated macrophages, cancer-associated fibroblasts, and cytotoxic lymphocytes all reshape the effective values of $J_{lf}$ and λ by remodeling ECM, secreting soluble factors, and exerting selective pressure on different invasion patterns [10,41,62]. While our model focuses on mechanical principles in in vitro assays, these principles may be relevant to understanding how multicellular escape strategies emerge in more complex tissue contexts [47,49]. Embedding our leader–follower mechanics into a richer microenvironment with stromal and immune agents is, therefore, a natural next step that may shift or fragment the current phase boundaries.

Despite these simplifications, the present work provides a high-resolution, mechanistically interpretable map from cell-level properties to tissue-level invasion patterns. Its principal contribution is not simply the identification of four phenotypes, but the demonstration that multimodal invasion emerges naturally within a broad mechanical regime and that cluster formation, finger formation, and single-cell escape are alternative consequences of the same adhesion-motility balance. The framework replaces a binary view of invasion with a connected landscape of mechanically accessible states and offers a reproducible basis for experiments designed to test how coordinated changes in cohesion and motility reorganize tumor invasion. By making the model and analysis code available and framing invasion control as movement on a phase diagram, we also aim to provide a reusable computational tool for designing experiments that can shift tumors away from cluster-forming, metastasis-prone states by jointly modulating cohesion and motility.

## Supporting information

S1 Fig Preliminary 3D simulations confirm that the 2D domain captures key structural features of tumor invasion. (TIF)

S2 Fig 3D volumetric heatmap and cross-sectional views of the invasive area across the full parameter space. (TIF)

S3 Fig Combined 3D and 2D visualization of the infiltrative area across the parameter space. (TIF)

S4 Fig Visualization of isolated single-cell escape events quantified across parameter slices and in 3D. (TIF)

S5 Fig 3D contour plots and cross-sectional views of the number of finger-like protrusions across the parameter space. (TIF)

S6 Fig Combined 3D and 2D visualization of detached cluster counts across the parameter space. (TIF)

S7 Fig Sensitivity analysis showing the relative contributions of adhesion, migration, and proliferation to invasion metrics via distance correlation and random forest importance. (TIF)

S8 Fig 2D cross-sections and orthogonal views of the 3D phenotype phase space. (TIF)

S9 Fig Invasion metrics across the full biophysical parameter space at three leader cell fractions (15%, 25%, and 35%). (TIF)

S10 Fig Cluster centroid trajectories with TrackID labeling at representative parameter combinations. (TIF)

S11 Fig Full cluster size distributions across migration coefficient and adhesion at fixed proliferative probability (*PP* = 0.9). (TIF)

S12 Fig Centroid displacement of simulated clusters as a function of cluster size across the motility parameter space at *PP* = 0.9. (TIF)

S13 Fig Leader cell fraction in clusters across parameter space and proliferative probability values. (TIFF)

S14 Fig Cluster metrics as a function of contact energy at fixed migration coefficient. (TIFF)

S15 Fig Persistence versus cluster size relationship across proliferative probability values. (TIFF)

S1 Table Parameter regions associated with emergent invasion phenotypes based on the 3D phenotype map. (XLSX)

S1 Appendix Statistical analysis methods including distance correlation, random forest importance, and SEM computation. (PDF)

S2 Appendix Cluster tracking and event detection algorithm including centroid matching, splitting, merging, and persistence definitions. (PDF)

S1 Video Temporal evolution of tumor invasion in 3D simulation from MCS 0–700. (MP4)

S2 Video Temporal evolution of the four invasion phenotypes. (A) No invasion. (B) Single-cell invasion. (C) Bulk collective invasion. (D) Multimodal invasion.
(MP4)

S3 Video Temporal evolution of the 3D phenotypic phase space from MCS 0–700. (MP4)

S4 Video Dynamic tracking of cluster splitting, merging, and dissolution events over the course of a representative simulation. (MP4)
