# Supplementary material for: Clusters, fingers, and singles: A mechanical landscape of tumor invasion
Source: PLoS Comput Biol. 2026 Sep 8;22(9):e1014747. doi: 10.1371/journal.pcbi.1014747 (PMC13568528; doi:10.1371/journal.pcbi.1014747)
Supplement: Supplementary file 1 [file pcbi.1014747.s017.pdf]

S1 Appendix.

Statistical Analysis

Parameter sensitivity was quantified using multiple complementary approaches. Pearson and Spearman rank correlations were computed between each parameter (J_{lf}, λ, PP) and each invasion metric (invasive area, infiltrative area, fingers, singles, clusters) to assess linear and monotonic relationships, respectively. Distance correlation was also computed to capture nonlinear dependencies that Pearson or Spearman methods might miss. Feature importance was assessed using a random forest regression model (RandomForestRegressor, scikit-learn 0.24) trained to predict all five invasion metrics simultaneously from the three input parameters. The model consisted of 400 decision trees, and feature importance was quantified as the mean decrease in impurity (Gini importance) across all trees, normalized to sum to 1. This approach provides a model-agnostic measure of each parameter's contribution to explaining variance in invasion outcomes.

Phase diagrams show the most frequent phenotype for each parameter combination, based on $n = 10$ replicates. For marginal analyses (Fig 3), parameters were binned into 11 levels corresponding to their discrete scan values, and invasion metrics were averaged within each bin. Error bars and shaded regions represent the standard error of the mean (SEM) unless otherwise noted. All statistical analyses were performed in Python 3.9 using NumPy, SciPy, and scikit-learn libraries.
