# Supplementary material for: Clusters, fingers, and singles: A mechanical landscape of tumor invasion
Source: PLoS Comput Biol. 2026 Sep 8;22(9):e1014747. doi: 10.1371/journal.pcbi.1014747 (PMC13568528; doi:10.1371/journal.pcbi.1014747)
Supplement: Supplementary file 2 [file pcbi.1014747.s018.pdf]

S2 Appendix.

Cluster Tracking, Event Detection, and Interactive Trajectory Explorer

Clusters were tracked across timepoints using a centroid-based nearest-neighbor matching algorithm to assign stable identities over the simulation trajectory. At each analysis timepoint (t, sampled every 10 MCS), cluster centroids were computed as the mean (x, y) coordinates of member cells. Clusters at timepoint $t + 1$ were matched to clusters at t by minimizing centroid displacement, subject to a maximum displacement threshold of 12 px (∼30 µm, corresponding to approximately three cell diameters). Clusters exceeding this threshold were assigned new identities, under the assumption that they represent newly formed aggregates rather than continuations of existing clusters.

Splitting events were identified when one cluster at time t matched multiple clusters at time $t + 1$, indicating fragmentation. Merging events were identified when multiple clusters at t matched a single cluster at $t + 1$, indicating coalescence. Cluster dissolution was identified when a cluster at time t had no matched descendants at $t + 1$, and its member cells were either absorbed into the main tumor or dispersed as singles. Overlap fractions for merges and splits were quantified using Jaccard similarity coefficients between member cell sets, thresholded at $J \geq 0.15$ to exclude spurious matches.

Cluster persistence was defined as the duration in MCS during which a cluster maintained continuous identity through the tracking algorithm, measured from first emergence to either dissolution or the simulation endpoint ($t = 700$ MCS). Clusters terminating before the simulation endpoint are distinguished from those persisting to the final time point to account for right-censoring in persistence estimates.

To enable interactive exploration of cluster centroid trajectories across the full parameter space, we developed a web-based supplementary interface available at: `https://sheriffacode.github.io/Cluster_Trajectory_Explorer/`

The explorer allows users to select any combination of leader fraction, leader–follower contact energy (J_{lf}), migration coefficient (λ), proliferative probability (PP), replicate index, and simulation timepoint (MCS), and displays the corresponding cluster centroid trajectories with full persistence and displacement information. Cluster termination status is encoded visually: a solid cross ($\times$) marks clusters that dissolved or reintegrated into the primary mass prior to the simulation endpoint, while a dotted cross marks clusters that persisted through the final timepoint at 700 MCS. This distinction allows readers to identify early-dissolving clusters separately from those whose full persistence history is censored by the finite simulation window.
