# Supplementary material for: Clusters, fingers, and singles: A mechanical landscape of tumor invasion
Source: PLoS Comput Biol. 2026 Sep 8;22(9):e1014747. doi: 10.1371/journal.pcbi.1014747 (PMC13568528; doi:10.1371/journal.pcbi.1014747)
Supplement: Supplementary file 3 [file pcbi.1014747.s024.pdf]

Response to Reviewers:

Clusters, Fingers, and Singles: A Mechanical Landscape of Tumor Invasion

Tracking #: PCOMPBIOL-D-26-00250

Sheriff Akeeb, Adam I Marcus, Yi Jiang

We sincerely thank all three reviewers for their careful reading and constructive feedback. These insightful critiques have substantially improved the clarity, rigor, and presentation of our manuscript. We have carefully addressed each comment and made significant revisions throughout. Below, we provide a detailed, point-by-point response to each reviewer's comments (quoted in black) with responses shown in red and revisions shown in blue in the manuscript.

REVIEW 1

In this work, Akeeb et al explore the emergence of different cancer invasion phenotypes using an agent-based model with explicit control of key parameters that determine the cells' proliferation rate, adhesion, and migration. The tumor has only two cell types, migrating (leader) and non-migrating (follower), where one of the latter is able to proliferate. Using a simplified 2D geometry of a non-small tumor boundary and a well-defined set of metrics, they tested many combinations of the 3 parameters (proliferation, migration, and leader-follower adhesion) and classified their results into 4 categories: non-invasion, single-cell invasion, fingering, and multimodal, with the latter being the dominant phenotype within their parameter space.

I found the manuscript to be very well written, with a clear introduction that summarizes the relevant points of the cancer literature in the context of their work, and very well-described methods and results. Their frequent reference and parallel of their simulation results with experimental observations is also a point that strengthens their manuscript. While I do have concerns about some specific aspects of the model, they are minor, and I think that they can be addressed easily. Most of my concerns/critiques relate to the structure of the paper and the lack of data for some of the results. For these reasons, I believe this manuscript should be revised before acceptance.

We sincerely thank the reviewer for the comprehensive and encouraging feedback. The positive assessments of our manuscript structure, methods description, and integration of experimental validation are greatly appreciated.

Comment 1: My main critique of their work is with the sequence of main figures, their choice of panels/graphs selected to be in the main vs. supplemental figures, and the lack of supporting data for some very interesting/relevant results that are only alluded to in the text. For example, I found

panels A, B, and C of Figure S9 more informative than Fig. 5 – the only thing missing would be the persistence time, but I think that this latter topic may deserve its own figure, or an extra panel.

We reorganized the cluster formation figures accordingly. Former Supplementary Figure S9 A-C has been moved to the main text as Figure 6 to directly visualize cluster incidence and composition across parameter space. Cluster size distributions are now presented in Figure 7, while the comprehensive cluster persistence time landscape remains in the Supplementary Materials. Corresponding figure references and narrative flow in the Results section were updated accordingly.

Comment 2: The whole section on cluster formation is full of very interesting results with no accompanying data or figure to back up or illustrate. On line 466, the authors claim that "centroid displacement increased with cluster size and motility", an interesting result, but there is no accompanying figure to help the readers visualize it. The same goes for the claims about the correlation of speed with cluster size and leader fraction. Three paragraphs later, the author mentions some interesting results about cluster splitting and merging events, but there are no figures accompanying that. Given the importance of clusters in cancer invasion (a point that the authors themselves emphasize) all these results deserve their own figure on the main text.

We agree that several statements regarding cluster dynamics required stronger quantitative support. We added Supplementary Figure S12 to visualize cluster centroid displacement and related dynamics. Because centroid displacement strongly correlates with persistence time, we now report this relationship explicitly in the revised text. We also clarified that cluster velocity is difficult to define uniquely because cluster identity changes through merging and splitting events during simulations. Splitting and merging dynamics are directly visualized in Supplementary Video S4 and can be explored interactively across all parameter combinations and replicates through the Interactive Cluster Trajectory Explorer described in S2 Appendix, where cluster termination status, dissolution before the endpoint versus persistence to the endpoint, is also encoded for each trajectory.

Comment 3: At the start of the Results section there is a lot of description about the role that each parameter has on the simulation results, but the graphs that illustrate those results only come in Fig 6. I do not understand the reasoning for placing that figure last. More information should be given about how those graphs were constructed.

Figure 6 has been moved to immediately follow Section 3.1 (now Figure 3). We also expanded the caption and Results text to explain how the metrics were constructed. Panels A–E show mean $\pm$ standard error of mean (SEM) for each invasion metric while varying one parameter at a time around baseline values, whereas Panel F summarizes phenotype frequencies across all simulations. The revised text now clarifies the binning and averaging procedure used to generate these plots.

Comment 4: A minor concern is the proliferation model itself. While it makes perfect sense that proliferation only scales the tumor size, but does not change the mode of invasion, I wonder if the results would be different if there was any sort of contact-inhibition of proliferation in the model. This can potentially change the results and might be worth discussing and/or considering as a future development of the model.

Contact-inhibition of proliferation is partially implicit in the Cellular Potts Model (CPM) because cells maintain finite target volumes and proliferation only occurs after sufficient volume growth, which becomes difficult in densely packed regions. We nevertheless agree that an explicit cell density-dependent proliferation rate would be an important future extension and could further strengthen adhesion-dependent effects on invasion phenotypes. This point has now been added to the Discussion.

Comment 5: Another minor concern that should be addressed is with regard to the measurements of persistence movement.Those measurements are not described in the methods, so the readers are left wondering what exactly the authors mean by it. I am also concerned about the end-of-simulation interruption of the persistent measurements. They are potentially biasing the results to shorter average persistence times and also giving a higher weight to simulations where clusters arise earlier.

Persistence time is now explicitly defined in the Methods as the interval between cluster emergence (first identified as a separate component via breadth-first search) and either dissolution (loss of cluster status or reintegration of all cells) or censoring at the simulation endpoint (700 MCS). We agree that endpoint censoring biases late-forming clusters toward shorter observed persistence times. However, 700 MCS represents approximately 36 hours of biological time, matching the experimental invasion assay duration. To address this concern transparently, we now separately report clusters that dissolved during simulation and clusters that persisted to the endpoint.

Comment 6: I also found it odd that there is a whole subsection at the end of the results section that is dedicated to talking about the lack of role of proliferation on the results. The point was already made clear at the start of the results section, and maybe it is better to expand on it at the beginning and leave the rest of the results section to focus on the more interesting results. This is just a suggestion, of course.

We removed the standalone subsection "Proliferation Plays a Limited Role" and instead integrated proliferation-related findings throughout the Results section. Section 3.1 now discusses proliferation effects on invasion metrics, Section 3.2 addresses phenotype classification, and Section 3.3 focuses on cluster dynamics. This reorganization reduces redundancy and better emphasizes the primary roles of adhesion and motility.

Comment 7: Line 232: BFS acronym mentioned before definition (on line 241)

Corrected. The BFS acronym is now defined immediately upon first use.

Line 232 revised to "... using a combination of breadth-first search (BFS), ..." The full definition now appears on first mention.

Comment 8: The two paragraphs on lines 231-249 repeat each other.

We have consolidated them into a single, more concise description.

The invasive and infiltrative areas have been consolidated into a single coherent paragraph. Redundant explanations have been removed, and the text now flows more naturally from the operational definitions to the biological interpretations.

Comment 9: Fig 2: There are too many elements in this figure, making it hard to understand/visualize all. My suggestion is to use a lighter tone for the follower cells and remove the special label for leader/follower on the cluster, which are very hard to distinguish from their counterparts. Also, the detected fingers label seems not to be present in the figure.

Thank you for this valuable feedback. We have implemented all three suggestions to improve the clarity of Figure 2. First, we changed follower cells to light green to reduce their visual dominance and redirect attention to the invasion boundaries. Second, we removed the special teardrop and lime-green markers for individual cells within clusters, keeping only the magenta X for cluster centroids. Third, we enabled the legend and marked detected boundary peaks (fingers) with yellow pentagon markers with navy edges, and implemented z-order layering to ensure they always appear

on top. These changes simplify the visualization while preserving all essential biological information.

Figure 2 was revised to use light green for follower cells, remove cluster member labels, and add yellow pentagon markers for boundary peaks with explicit legend labels and z-order layering for visibility. The figure caption was updated to reflect these changes, specifying the use of light green for follower cells, yellow pentagons with navy edges for boundary peaks, and explicitly listing all annotated features.

REVIEW 2

This is a comprehensive computational study on how tumors invade surrounding tissue given different physical characteristics. The paper builds a computer simulation of differing adhesion, motility, and proliferation using the Cellular Potts Model. They ran 1331 different combinations of three key parameters, with 10 replicates each. The authors identified four invasion phenotypes (distinct behavioral modes), and this paper links back to biology to refer to key tumor invasion dynamics known in the literature. Below are the remaining concerns with the paper that the authors should address:

We thank the reviewer for their thorough and insightful assessments.

Comment 1: The authors should justify the rule-based classifier, as the outcome of the phenotypes depends entirely on these thresholds. This could also be addressed as a sensitivity analysis or validated against manually labeled snapshots.

This is a valid methodological concern. The classifier thresholds are biologically motivated and distinguish non-invasive growth, solitary escape, cohesive fingering invasion, and mixed multimodal invasion. Representative simulations in Figure 2 visually validate these phenotype assignments. To further assess robustness, we added a sensitivity analysis in which key classifier thresholds were varied by ±20%. Phenotype boundaries shifted smoothly and overall classifications remained stable across perturbations.

We added to the Methods section: "Sensitivity analysis on classifier thresholds (±20% variation on finger prominence, defector cutoffs, and area ratios) confirmed phenotype classification robustness, with stable phenotype boundaries across threshold perturbations.

Comment 2: The fixed leader fraction should be explored, but is labeled as future work. The leader fraction is also stated as 30% in other studies. Did the authors consider running 2 condition comparisons on either side of 25%, e.g., 15 and 35%, to show that this value is robust? I understand that computational resources might prevent a full run on each of these 3 (15, 25, 35%) leader fractions.

Thank you for this insightful suggestion. We have now performed the requested bracketing analysis by running full simulations at leader fractions of 15%, 25%, and 35%, spanning the range on either side of our original choice. Supplementary Fig S9 shows all six invasion metrics across the complete three-dimensional parameter space for each condition, with color scales unified within each metric to enable direct cross-condition comparison.

The results support the robustness of the invasion metrics against leader fraction. The infiltrative area and the number of detached single cells increase monotonically and linearly with the leader fraction. Finger-like protrusions follow the same trend but saturate between $LF = 0.25$ and

LF = 0.35, consistent with a geometric constraint on simulation size. Cell clusters exhibit an inverse relationship with leader fraction, reflecting a biologically coherent transition from cohesive to individual detachment as the number of leader cells increases. Critically, the rank ordering of parameter effects and the structure of the phenotype phase space are conserved across all three leader fractions.

The choice of 25%, therefore, sits within a regime where results are representative of the broader range and is directly supported by the experimentally reported value of approximately 30% in NSCLC spheroids [1].

Together with the new Supplementary Figure S9, we have added these new results in Results and discussed the role of leader fraction in Discussion.

Comment 3: Proliferation is not a primary determinant of invasion mode, but it does regulate metastatic potential by controlling cluster size and persistence. "Proliferation Plays a Limited Role" should be reworded to reflect this nuance.

We agree and have revised the Results organization accordingly. The revised manuscript now distinguishes proliferation's limited role in determining invasion phenotype from its important effects on tumor mass, cluster size, and cluster persistence.

Comment 4: In addition, please address repeated sections in lines 234-239 and 254-258. They seem to convey the same information.

We have consolidated them as addressed above in Response to Reviewer 1.

REVIEW 3

This manuscript by Akeeb et al investigates how the Cellular Potts model generates various forms of cell escape from a cellular mass representing the edge of the tumor. By varying three different model parameters, the authors formulate hypotheses of which mechanical properties of the cells are responsible for distinct patterns of cell migrations out of the tumor edge. The results are interesting and within the scope of the PLOS Computational Biology journal. I would recommend this manuscript for publication, provided the critiques listed below are addressed.

We sincerely thank the reviewer for the positive assessments and comprehensive evaluation. The detailed critiques have significantly strengthened the manuscript by clarifying our model assumptions and improving the accessibility of our framework.

Comment 1: The authors should be more precise about what is actually modeled. As the authors mentioned in the introduction, about tumor invasion "... local invasion, where tumor cells breach the basement membrane and infiltrate surrounding tissues". The presented model has no basement membrane, and no organ structure that the tumor is growing in – the lung for NSCLC; thus, the title and model's results should indicate that this is a model of the in vitro system.

Thank you for the important observation about the distinction between in vivo tissue invasion and our simplified in vitro model. We revised the manuscript to clarify that the model represents a simplified in vitro invasion assay rather than in vivo tissue invasion. The framework intentionally excludes basement membrane structure, stromal interactions, and organ-scale heterogeneity to isolate the mechanical effects of adhesion and motility. These limitations and their implications for translation to in vivo systems are now stated explicitly in the Introduction, Methods, and

Discussion.

The Introduction, Methods, and Discussion sections have been revised to explicitly clarify that the model represents a simplified in vitro invasion assay without a basement membrane, organ tissue context, or stromal microenvironment. The Discussion limitations section now emphasizes that translation to in vivo invasion requires coupling these principles with additional in vivo complexity.

Comment 2: The invasion should be defined. How far from the egg of the tumor mass must the cells be to be considered invasive?

In our model, invasion is operationally defined by the morphological transition from a smooth, compact tumor boundary to one exhibiting protrusions or cellular detachment from the core tumor mass. As described in the Model and Methods: "A protruding figure is defined as a prominence ≥ 10 pixels in the y-direction (corresponding to $\approx 30\mu m$) protrusion height, separation of at least 20 pixels ($\approx 60\mu m$) from neighboring peaks in the x-direction, and width at half-prominence ≥ 5 pixels."

Comment 3: The computational model should be defined in the main text, not relegated to the Supportive material.

We agree and have moved the Model and Methods section into the main text. This includes the complete Cellular Potts Model framework (energy functional and Metropolis algorithm) and all computational implementation details.

Comment 4: In the Friedl 2012 paper, there are 8 different patterns of individual/collective cell migration identified. Were all these patterns achieved by the CP model in this manuscript?

We thank the reviewer for this excellent point. Friedl *et al.* 2012 organized invasion into patterns across four hierarchical categories: single-cell modes (amoeboid and mesenchymal), multicellular streaming, collective migration, tissue folding, and expansive growth. Our model captures the biological essence of most of these, though not always as explicitly named categories. Single-cell amoeboid and mesenchymal modes both appear as detached solitary cells in our simulations but are unified under "Singles" because their mechanistic distinction depends on explicit cell-ECM proteolysis absent from our framework. Multicellular streaming emerges under high migration and weak adhesion, corresponding to both streaming variants, again unified under our "Singles" and "Multimodal" categories for the same reason. Finger formation in our Bulk and Multimodal phenotypes directly corresponds to the mesenchymal and epithelial collective modes, and proliferation-driven expansion in the "No Invasion" phenotype captures expansive growth precisely. The one pattern genuinely absent is tissue folding, which requires asymmetric actomyosin contraction driving passive tissue-level deformation, incompatible with our unidirectional chemotactic gradient. These limitations are consistent with our deliberate focus on isolating the mechanical consequences of adhesion and motility in a simplified in vitro framework, as discussed in the limitations section.

We have revised the Discussion to explicitly acknowledge that our model captures several invasion modes described by Friedl et al., including solitary escape, multicellular streaming, collective fingering, and expansive growth. However, distinctions requiring explicit ECM proteolysis or tissue architecture are not resolved in the present framework, and tissue-folding modes lie outside the scope of the model. We now clarify these correspondences and limitations in the Discussion.a mechanistic subset of the Friedl invasion spectrum, noting that some patterns requiring explicit cell-ECM proteolysis or luminal organization are present morphologically but not formally separated, and that tissue folding is the one pattern outside the model's scope.

Comment 5: Fig.3 – the details of these simulations are not visible, such as the single cell. A white background may be better for the visibility of the results.

We thank the reviewer for this observation. We have updated Figure 3 with improved visibility by adopting a clear background and a brighter color scheme. Leader cells are now represented in red, follower cells in green, and detached clusters in cyan, all of which provide enhanced contrast against the light background. The revised figure now clearly shows the single cells, fingers, and clusters across all phenotypes, making the morphological distinctions more apparent at each time point.

We replaced Figure 3 with an updated version featuring a light background and enhanced color contrast.

Comment 6: Fig.4A – This 3D parameter space should be drawn transparent to show the shapes of the subspaces on the back side.

We explored transparent renderings of the parameter space following the reviewer's suggestion. However, transparency substantially reduced visual separation between the Bulk and Multimodal regions because of overlapping warm color tones. We therefore retained the opaque rendering for clarity, while Supplementary Video S3 and Supplementary Figure S8 provide rotating and sectional views that expose the internal phase structure more effectively.

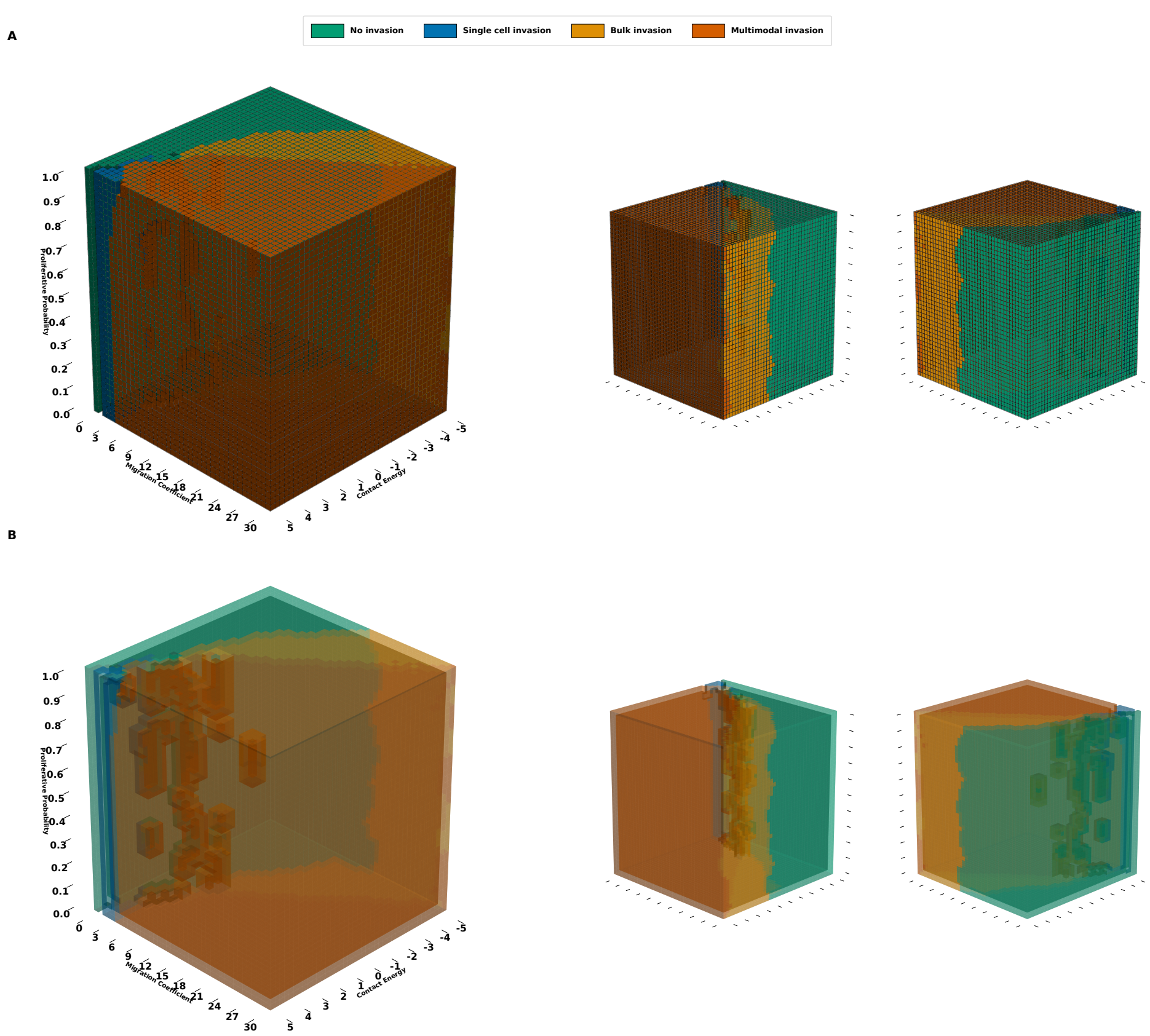

Figure. Opaque versus transparent renderings of the 3D phenotype parameter space. (**A**) Opaque rendering as presented in Fig. 4A, shown from four viewing angles. (**B**) Transparent rendering (surface-shell only, $\alpha = 0.40$). Phenotypes are color-coded as: green (No Invasion), blue (Single-Cell Invasion), orange (Bulk Invasion), and gold (Multimodal Invasion).

The supplementary video S3 Video shows a rotating view of the full parameter space, and Sup-

plementary Fig S8 shows 2D cross-sections. Together, they provide the 3D views of the phase diagram.

Comment 7: How does the 3D parameter space in Fig.4A compare to the 3D parameter space of cell migration from the Friedl 2012 paper that the authors cited?

This is a perceptive observation. While both frameworks use three-dimensional parameter spaces to organize invasion phenotypes, they differ in their parameterization. Friedl et al. (2012) organized invasion modes around morphological and polarity descriptors: leading-edge polarity, cell-cell contacts, and apicobasal polarity. Our Fig. 4A organizes phenotypes by the mechanical parameters that control their emergence: adhesion strength, leader cell motility, and follower cell proliferation. This difference reflects our focus on the mechanistic drivers of invasion mode transitions in a simplified two-cell-type system with uniform ECM. Both spaces are complementary: Friedl's framework describes the morphological and organizational phenotypes observed across diverse biological systems and tumor types, while our parameter space identifies the minimal mechanical conditions necessary to generate phenotypic transitions in silico. The correspondence between our mechanical parameters and Friedl's descriptors (e.g., strong adhesion supporting cohesive groups, high motility enabling single-cell escape, balanced proliferation enabling intermediate modes) demonstrates that our model captures mechanistically consistent behavior aligned with established invasion taxonomy.

Comment 8: Fig.4 – the majority of simulated patterns are multimodal. Is this pattern observed in biology?

Yes, indeed. Multimodal invasion (simultaneous fingers, singles, and clusters) is increasingly observed in aggressive clinical tumors [2, 3]. More broadly, the Friedl framework emphasizes that tumor invasion is fundamentally heterogeneous, with most invasive carcinomas displaying mixed or unstable phenotypes rather than single invasion modes [4]. This invasion heterogeneity and phenotypic plasticity extend across multiple cancer types and represent a hallmark of aggressive disease [5]. Studies of collective migration in mammary tissue and fibroblast-led invasion similarly document the coexistence of multiple invasion strategies within single tumors. The prevalence of multimodal invasion in our simulations, therefore, aligns with the established view that tumor cells employ heterogeneous and context-dependent invasion strategies.

Comment 9: Section "Cluster formation peaks ..." nine of the ten references are to three Supplemental figures. If these figures are necessary for understanding the results, they should be included in the main text.

Thank you for the suggestion. We have addressed this concern by promoting the critical cluster formation figures to the main text. Figure 6 displays cluster incidence, size distribution histograms, and composition data, which are essential for understanding cluster formation propensity and cellular organization. Figure 7 shows detailed cluster size distributions across the parameter space. These two main figures now provide a more complete visualization of cluster behavior. The remaining supplementary references in the cluster formation section are for secondary mechanistic details: cluster trajectories and velocities, comprehensive persistence landscapes, and dynamic splitting/merging events, which enhance understanding of the core cluster formation mechanisms presented in the main text.

The revised cluster formation section now includes Figures 6 and 7 as core results, with supplementary figures for more detailed analyses that support it.

Comment 10: Comparison between cells invading the tissue/matrigel with CTCs is not appropriate, CTC transport is in a completely different microenvironment.

This is an excellent and important clarification. We agree that circulating tumor cell clusters (CTCs) exist in a fundamentally distinct microenvironment from the in vitro invasion assays our model represents. Our model cannot and does not capture critical in vivo factors. The mechanical principles we identify (leader-mediated cohesion and intermediate adhesion favoring cluster stability) may be relevant to understanding cluster persistence in experimental assays, but direct extrapolation to CTC metastatic efficiency is inappropriate without explicit modeling of the vascular microenvironment.

We revised the manuscript to remove the direct comparison between the simulated cluster sizes and CTC metastatic efficiency. The revised passage now states that our cluster size range is consistent with experimental invasion assays and that the mechanical principles identified may inform future investigation of cluster stability in vivo, but we explicitly acknowledge that CTCs operate in a distinct microenvironment and do not make claims about CTC metastatic behavior based on our in vitro model.

Comment 11: Most of the presented results are quite obvious: invasion is dictated by migration, depends on weak adhesion, and not on proliferation. Is there any unexpected result that was generated by the model?

While some individual parameter effects are intuitive, the novelty of this work lies in the systematic exploration of their coupled nonlinear interactions. Several findings were not obvious a priori: (1) finger formation is maximized at intermediate rather than maximal adhesion, (2) multimodal invasion dominates much of parameter space, contradicting binary invasion models, (3) detached clusters are leader-enriched rather than passive fragments, and (4) proliferation alters tumor mass and cluster persistence without substantially changing invasion phenotype. Together, these results generate quantitative and experimentally testable predictions regarding how joint modulation of adhesion and motility reshapes invasion behavior.

The 3D phase map provides quantitative, testable predictions for how joint modulation of adhesion and motility can shift tumors between invasion modes, distinguishing this work from prior studies that examined individual parameters in isolation.

Comment 12: It seems that followers-only clusters are allowed. How can the followers only clusters separate from the main tumor mass, if there are no leaders in the cluster? Aren't the followers following the leader cells?

This is a very good observation. Follower-only clusters arise through cluster splitting events where mixed-composition aggregates (leaders and followers) fragment during the simulation, creating daughter clusters that may contain only followers. These clusters lack active motility and exhibit negligible displacement (approximately zero net movement) compared to leader-enriched clusters. They are rare and short-lived relative to leader-dominated aggregates (which peak at approximately 60 percent leader fraction). Thus, they represent transient passive fragments rather than active escape units, and their presence reinforces that invasion coordination depends on leader cell presence.

We clarify that follower-only clusters are rare, lack motility due to the absence of leaders, and persist much shorter than leader-enriched clusters.

Comment 13: Table 1 should contain parameters with physical units and references to biological literature that is a source of experimental values that agree with the values chosen for parameters.

We have revised Table 1 to include superscript notation (i–iv) indicating the basis for each parameter value. The expanded caption explains that superscript (i) denotes parameters calibrated to experimental data, (ii) denotes parameters selected from preliminary parametric exploration, (iii) denotes parameters based on literature values or experimental observations, and (iv) denotes standard CPM framework parameters. A detailed "Parameter Selection Rationale" paragraph below the table explains which parameter ranges were explored and why the refined ranges were selected, providing biological justification and literature references for each parameter choice. This ensures full transparency in how parameters were determined and validated.

Table 1 now includes superscript notation (i–iv) in the "Model Value" column identifying the source basis for each parameter. Range values are formatted in brackets [a, b] for clarity (e.g., [0, 30] for migration coefficient, [-5, +5] for contact energy). The table caption explains each superscript category. A comprehensive "Parameter Selection Rationale" paragraph immediately below the table describes: (1) calibration of leader migration speed to experimental velocities from Konen et al. 2017, (2) selection of J_{lf} range from broader [-10, +10] exploration after testing, (3) justification for leader cell fraction (25%) matching NSCLC observations, (4) proliferation rates matching aggressive cancer cell lines, (5) target cell volume calibration to carcinoma cell diameter, and (6) explanation of contact energy choices for other cell type pairs.

Comment 14: How can the model be calibrated to experimental data? What should be measured experimentally to fit the J parameters?

This is an important methodological question. We have partially calibrated the model by matching leader cell migration velocities to experimental measurements from 3D collagen-based invasion assays (Methods section), achieving agreement between simulated velocities (0.9-1.2 micrometers per minute at high lambda) and experimental observations (1.1 ± 0.3 micrometers per minute). This calibration anchors the temporal and spatial scales of the model.

However, direct calibration of the J_{lf} parameter (adhesion) to experimental data is fundamentally challenging because intercellular adhesion strength is not measured as a single quantity. Rather, adhesion emerges from the combined effect of cadherin expression levels, cadherin type ratios (E-cadherin versus N-cadherin), junction maturity, and mechanical properties of the underlying cytoskeleton. No single biomarker directly predicts J_{lf} values. Moreover, these factors interdepend with motility and other cellular properties, complicating direct parametrization.

Despite this limitation, our approach provides biological interpretability. The Discussion establishes that J_{lf} maps onto cadherin balance (low J_{lf} corresponds to high E-cadherin, strong adhesion; high J_{lf} corresponds to N-cadherin predominance, weak adhesion). Experimentally, this can be approximated through immunofluorescence quantification of E-cadherin and N-cadherin levels, or through measurements of junction stability using live-cell imaging of cadherin dynamics. Similarly, lambda can be estimated from organoid assays by measuring leader cell velocity and persistence, while proliferative probability can be quantified using Ki67 staining.

The phase diagram itself provides a framework for inverse calibration: by measuring these properties in experimental tumors and estimating their position in parameter space, one can predict invasion phenotypes and test whether the model correctly predicts observed morphologies. This validation-through-prediction approach offers a practical path to calibration without requiring direct measurement of effective contact energies.

We describe in the Discussion how proxy experimental measurements (cadherin ratios, junction dynamics, leader velocity, Ki67 proliferation markers) can constrain parameter estimates and enable model validation through phenotype prediction.

References

[1] Khatib TO, Pedro BA, Bombin S, Matsuk VY, Robinson IE, Webster SF, et al. TGF-β1-mediated intercellular signaling fuels cooperative cellular invasion. Cell Reports. 2025;44(2). doi:10.1016/j.celrep.2025.115315.

[2] Sahoo S, Ramu S, Nair MG, Pillai M, San Juan BP, Milioli HZ, et al. Increased prevalence of hybrid epithelial/mesenchymal state and enhanced phenotypic heterogeneity in basal breast cancer. Iscience. 2024;27(7). doi:10.1016/j.isci.2024.110116.

[3] Cheung KJ, Ewald AJ. A collective route to metastasis: Seeding by tumor cell clusters. Science. 2016 April;352(6282):167-9. doi:10.1126/science.aaf6546.

[4] Friedl P, Locker J, Sahai E, Segall JE. Classifying collective cancer cell invasion. Nature cell biology. 2012;14(8):777-83. doi:https://doi.org/10.1038/ncb2548.

[5] Friedl P, Alexander S. Cancer invasion and the microenvironment: plasticity and reciprocity. Cell. 2011;147(5):992-1009. doi:10.1016/j.cell.2011.11.016.
