# Supplementary material for: Clusters, fingers, and singles: A mechanical landscape of tumor invasion
Source: PLoS Comput Biol. 2026 Sep 8;22(9):e1014747. doi: 10.1371/journal.pcbi.1014747 (PMC13568528; doi:10.1371/journal.pcbi.1014747)
Supplement: Supplementary file 4 [file pcbi.1014747.s025.pdf]

Response to Reviewers (Second Round Revision): Clusters, Fingers, and Singles: A Mechanical Landscape of Tumor Invasion

Tracking #: PCOMPBIOL-D-26-00250R1

Sheriff Akeeb, Adam I Marcus, Yi Jiang

We sincerely thank the editors and reviewers for their continued engagement with our work. We are pleased that the reviewers found the revised manuscript substantially addressed the major concerns raised in the initial review. We have carefully considered the remaining comments from Reviewer 1 and the suggestions from Reviewer 3 and have made targeted revisions to improve the clarity, accuracy and presentation of the manuscript.

Below, we provide a point-by-point response to each remaining comment.

REVIEW 1 - ADDITIONAL COMMENTS

We thank Reviewer 1 for the encouraging overall assessment and for identifying several remaining areas to improve figure clarity and data presentation.

Comment 1: The lack of clear supporting data/figures for claims about cluster leader fraction and persistence time. Paragraphs starting at line 519 and references to figures S11 and S12 lack clear figures/data support. Figures S11 and S12 are very hard to read and understand.

We appreciate this concern and have added three new supplementary figures to provide clearer and more direct support for the conclusions regarding cluster composition and persistence. S13 Fig maps leader cell fraction across the adhesion-migration parameter space at four proliferation probabilities and reports the number of observed clusters in each parameter bin. S14 Fig summarizes cluster persistence time, leader cell enrichment, and mean cluster size distribution as a function of contact energy, with error bars showing variability across simulations. S15 Fig relates cluster size to persistence time across proliferation probabilities using hexagonal-binning plots.

We have also clarified the reference in the inset panels of S11 Fig. These insets now directly support the discussion about heterogeneous cluster outcomes by showing that small clusters (4–8 cells) have shorter persistence times than rare, large aggregates at specific parameter combinations (e.g., $\lambda = 30, J_{lf} = 2$). We further clarify that because most of the clusters live till the end point of the simulation, the longer persistence time corresponds to earlier cluster formation.

We emphasize that summarizing cluster dynamics across 13 310 simulations with multiple parameter dimensions and heterogeneous cluster dynamics presents substantial visualization challenges. To improve transparency and allow readers to explore the results beyond the static figures, we

have made the raw cluster data available on GitHub (`https://github.com/Jiang-Lab/Tumor_Invasion_Model`) and created an Interactive Cluster Trajectory Explorer (`https://sheriffacode.github.io/Cluster_Trajectory_Explorer/`). This tool enables readers to examine individual cluster trajectories, splitting/merging events, and persistence patterns across the parameter space.

Comment 2: Line 112: Repeated information. Better to replace that sentence with an explanation of what an MCS is.

We agree and have replaced the redundant description of CompuCell3D with the following definition of Monte Carlo Step (MCS): "One Monte Carlo Step (MCS) comprises a number of attempted lattice-site copy events equal to the total number of lattice sites, so that each lattice site is selected once on average. Simulations were run for 700 MCS, corresponding to approximately 36 h of biological time."

Comment 3: Line 124: "Simplified" appears twice.

Fixed.

Comment 4: Lines 360-361: There are two mean number of finger values—are they with respect to intermediate adhesion only and strong migration only?

Yes. These values are marginal means calculated with respect to different model parameters. The first value is averaged across all migration and proliferation probabilities at intermediate adhesion, and the second is averaged across all adhesion and proliferation values under strong migration. We have revised the text to make this averaging procedure explicit.

The text now reads: "Finger counts peaked at intermediate adhesion ($J_{lf} \approx 0$ to 2), yielding a marginal mean of 7.11 fingers (averaged across all migration coefficients and proliferative probabilities), and at strong migration ($\lambda \geq 20$) with a marginal mean of 9.74 fingers (averaged across all adhesion strengths and proliferative probabilities).

Comment 5: Lines 405-407: "The most consistent effect of PP was on the overall invasive area, which increased under high proliferation..." This trend is not visible in Fig 3A. The PP lines appear to actually decrease.

We agree with the reviewer. Figure 3A does not support a consistent increase in invasive area with increasing proliferative probability. The sensitivity analysis instead shows that the association between (P_P) and invasive area is negligible, with Pearson $r < 0.03$. We have now removed the misleading statement and revised the corresponding Results paragraphs to describe the role of proliferation more accurately. The revised text explains that changes in proliferative probability produce nearly flat invasive-area curves, with only minor deviations across the tested range. We further clarify the mechanistic interpretation: high proliferation increases cellular crowding within the tumor core, which can restrict leader-driven protrusive extension of the connected invasion front while promoting cell detachment and cluster formation. Thus, proliferation may increase total tumor cell number or tumor bulk without necessarily increasing the spatial extent of the connected invasive front. This distinction is now stated explicitly in the manuscript.

Comment 6: Line 407: This sentence seems more appropriate in the figure caption.

We agree and have removed the sentence from the main text, as this technical detail is already

appropriately described in the Figure 3 caption.

Comment 7: Lines 508-509: No data/figure referenced to support observation.

We have added explicit figure references and revised the text to avoid implying leader dominance. Cluster composition is now referenced to Fig 6D,E, and the leader-fraction landscape is referenced to S13 Fig. The revised text states that clusters were generally enriched for leaders relative to the 25% initial leader fraction, with leader fractions commonly reaching approximately 60-70% in the cluster-forming regime. We no longer state that leaders outnumber followers in every cluster or use a single peak value across figures with different summaries.

Comment 8: Lines 345-350 and 356-357: The px^2 numbers carry no meaning by themselves. Suggestions: convert to $micrometers^2$ or speak in terms of fold or % increases/decreases with respect to other scenarios.

We agree and have systematically replaced area measurements in pixels squared with square micrometers throughout the manuscript. Where appropriate, we now also report fold-changes to convey the magnitude of the effects more clearly. The calibration reference is provided in the Methods section: "One pixel corresponds to approximately 2.5 µm, therefore, 1 $px^2 \approx 6.25$ μm^2. "

REVIEW 2

We thank Reviewer 2 for confirming that our revisions have adequately addressed the concerns raised in the initial review.

REVIEW 3

We thank Reviewer 3 for this helpful suggestion. We have substantially revised the Discussion to foreground the study's principal novel and non-intuitive findings. We now highlight that: (i) multimodal invasion, rather than a purely collective or solitary mode, occupies the largest region of the sampled parameter space; (ii) invasion architecture is governed primarily by the nonlinear interaction between leader-cell motility and leader-follower adhesion, whereas follower proliferative probability has little influence on phenotype classification over the simulated interval; (iii) detached clusters emerge within an intermediate-adhesion/high-motility window and are generally enriched for leader cells relative to the 25% initial leader fraction; and (iv) cluster outcomes are heterogeneous, with many small clusters and rare larger aggregates in the longer-recorded-duration tail. We have also clarified that cluster persistence is right-censored because most clusters remain present at the simulation endpoint, so longer recorded duration often reflects earlier formation rather than a fully observed intrinsic lifetime. Finally, we have tempered the translational language and now present the phase diagram as a source of experimentally testable hypotheses rather than as a direct therapeutic prescription.
