# Supplementary material for: Clusters, fingers, and singles: A mechanical landscape of tumor invasion
Source: PLoS Comput Biol. 2026 Sep 8;22(9):e1014747. doi: 10.1371/journal.pcbi.1014747 (PMC13568528; doi:10.1371/journal.pcbi.1014747)
Supplement: Peer review history

# Peer review: Decision Letter 0

**DOI:** 10.1371/journal.pcbi.1014747.r001

**Authors:** Dimitrios Vavylonis, Alex Tam

25 Mar 2026

Clusters, Fingers, and Singles: A Mechanical Landscape of Tumor Invasion

PLOS Computational Biology

Dear Dr. Jiang,

Thank you for submitting your manuscript to PLOS Computational Biology. After careful consideration, we feel that it has merit but does not fully meet PLOS Computational Biology's publication criteria as it currently stands. Therefore, we invite you to submit a revised version of the manuscript that addresses the points raised during the review process.

Please submit your revised manuscript by May 25 2026 11:59PM. If you will need more time than this to complete your revisions, please reply to this message or contact the journal office at ploscompbiol@plos.org. When you're ready to submit your revision, log on to https://www.editorialmanager.com/pcompbiol/ and select the 'Submissions Needing Revision' folder to locate your manuscript file.

Please include the following items when submitting your revised manuscript:

* A letter that responds to each point raised by the editor and reviewer(s). You should upload this letter as a separate file labeled 'Response to Reviewers'. This file does not need to include responses to formatting updates and technical items listed in the 'Journal Requirements' section below.

* A marked-up copy of your manuscript that highlights changes made to the original version. You should upload this as a separate file labeled 'Revised Manuscript with Track Changes'.

* An unmarked version of your revised paper without tracked changes. You should upload this as a separate file labeled 'Manuscript'.

If you would like to make changes to your financial disclosure, competing interests statement, or data availability statement, please make these updates within the submission form at the time of resubmission. Guidelines for resubmitting your figure files are available below the reviewer comments at the end of this letter

We look forward to receiving your revised manuscript.

Kind regards,

Alex Tam

Academic Editor

PLOS Computational Biology

Dimitrios Vavylonis

Section Editor

PLOS Computational Biology

**Journal Requirements:**

If the reviewer comments include a recommendation to cite specific previously published works, please review and evaluate these publications to determine whether they are relevant and should be cited. There is no requirement to cite these works unless the editor has indicated otherwise.

1) Please ensure that the CRediT author contributions listed for every co-author are completed accurately and in full.

At this stage, the following Authors/Authors require contributions: Sheriff Akeeb, Adam I Marcus, and Yi Jiang. Please ensure that the full contributions of each author are acknowledged in the "Add/Edit/Remove Authors" section of our submission form.

The list of CRediT author contributions may be found here: https://journals.plos.org/ploscompbiol/s/authorship#loc-author-contributions

2) We ask that a manuscript source file is provided at Revision. Please upload your manuscript file as a .doc, .docx, .rtf or .tex. If you are providing a .tex file, please upload it under the item type u2018LaTeX Source Fileu2019 and leave your .pdf version as the item type u2018Manuscriptu2019.

3) Your manuscript is missing the following sections: Methods.  Please ensure all required sections are present and in the correct order. Make sure section heading levels are clearly indicated in the manuscript text, and limit sub-sections to 3 heading levels. An outline of the required sections can be consulted in our submission guidelines here:

https://journals.plos.org/ploscompbiol/s/submission-guidelines#loc-parts-of-a-submission

4) Please upload all main figures as separate Figure files in .tif or .eps format. For more information about how to convert and format your figure files please see our guidelines:

https://journals.plos.org/ploscompbiol/s/figures

5) We have noticed that you have uploaded Supporting Information files, but you have not included a list of legends. Please add a full list of legends for your Supporting Information files after the references list.

**Reviewers' comments:**

Reviewer's Responses to Questions

**Comments to the Authors:**

**Please note here if the review is uploaded as an attachment.**

Reviewer #1: In this work Akeeb et al. explore the emergence of different cancer invasion phenotypes using an agent-based model with explicit control of key parameters that determine the cells proliferation rate, adhesion and migration. The tumor has only two cell types, migrating (leader) and non-migrating (follower), where one the latter are able to proliferate. Using a simplified 2D geometry of a non-small tumor boundary, and a well-defined set of metrics, they tested many combinations of the 3 parameters (proliferation, migration, and leader-follower adhesion) and classified they results into 4 categories: non-invasion, single-cell invasion, fingering and multimodal, with the latter being the dominant phenotype within their parameter-space.

I found the manuscript to be very well written, with a clear introduction that summarizes the relevant points of the cancer literature in the context of their work, and very well described methods and results. Their frequent reference and parallel of their simulation results with experimental observations is also a point that strengthens their manuscript. While I do have concerns about some specific aspects of the model, they are minor and I think that can be addressed easily. Most of my concerns/critiques relate to the structure of the paper and the lack of data for some of the results. For these reasons I believe this manuscript should be revised before acceptance.

My main critique of their work is with the sequence of main figures, their choice of panels/graphs selected to be in the main vs. supplemental figures, and the lack of supporting data for some very interesting/relevant results that are only alluded in the text. For example, I found panels A, B and C of figure S9 more informative than Fig. 5 -- the only thing missing would be the persistence time, but I think that this latter topic may deserve its own figure, or an extra panel.

The whole section on cluster formation is full of very interesting results with no accompanying data or figure to backup or illustrate. On line 466 the authors claim that “centroid displacement increased with cluster size and motility”, an interesting result, but there is no accompanying figure to help the reads visualize it. The authors only offer Fig S10, which is illustrates a single simulation and as such is more suggestive than demonstrative. In my view the authors should show something like a scatter plot of displacement vs cluster sizes and lambda. The same goes for the claims in the same paragraphs about the correlation of speed with cluster size and leader fraction. Three paragraphs later the author mention some interesting results about cluster splitting and merging events, but there is no figures accompanying that. Given the importance of clusters in cancer invasion (a point that the authors themselves emphasize) all these results deserve their own figure on the main text.

At the start of the Results section there is a lot of description about the role that each parameter has on the simulation results, but the graphs that illustrate those results only come in Fig 6. I do not understand the reasoning for placing that figure last. The figure by itself is a very good one, but there is no information about how the 2 other parameters were set while varying one. For example, on panel A, as you vary J, what are the values of lambda and PP? How did you choose them? I can tell it is not the middle value within their ranges, as this should make all curves cross at the 0.5 normalized parameter value mark, but this only happens in panel B. More information should be given about how those graphs were constructed.

A minor concern is the proliferation model itself. While it makes perfect sense that proliferation only scales the tumor size, but does not change the mode of invasion, I wonder if the results would be different if there was any sort of contact-inhibition of proliferation in the model. This can potentially change the results and might be worth discussing and/or considered as a future development of the model.

Another minor concern that should be addresses is with regards to the measurements of persistence movement. Those measurements are not described in the methods, so the readers are left wondering what exactly the authors mean by it. I am also concerned about the end-of-simulation interruption of the persistent measurements. They are potentially biasing the results to shorter average persistence times and also giving a higher weight to simulations where clusters arise earlier.

I also found odd that there is a whole subsection at the end of the results section that is dedicated to talk about the lack of role of proliferation on the results. The point was already made clear at the start of the results section, and maybe it is better to expand on it at the beginning and leave the rest of the result section to focus on the more interesting results. This is just a suggestion, of course.

Specific comments:

Line 232: BFS acronym mentioned before definition (on line 241)

The two paragraphs on lines 231-249 repeat each other.

Fig 2: There are too many elements in this figure, it is hard to understand/visualize all. My suggestion is to use a lighter tone for the follower cells and remove the special label for leader/follower on cluster, which are very hard to distinguish from their counterparts. Also, the detected fingers label seems to be not present in the figure.

Table 1: The varied parameter would be more visible if the asterix (*) came before their name. Another suggestion is to use (min,max) to display ranges in place of min – max, as the use of negative values might cause confusion.

Lines 264-274: the description of the finger detection algorithm is not clear. What is meant by a prominence of 10 pixels? What is the reference point from which this is counted? What is a half-prominence width? I think this whole section can be better described.

Line 277-8: “… and was located above the minimum y-coordinate of the main tumor”. Is it even possible to have a stranded cell below that level? I would think the tissue is confluent.

Lines 337-40: The px◯2 number carry no meaning by themselves. My suggestions are to either convert this to micrometers◯2 (which by itself also has not much meaning as it depends on how wide the simulation is) or speak in terms of fold or % increases/decreases with respect to other scenarios.

Lines 413-6: The description of the Single Cell Invasion do not match what is in Fig. 4.

Fig. 5: I found the x-axis of those graphs confusing. It took me a while to understand that it is a cumulative number of clusters for that simulation, where each bar indicate a single cluster, with its heigh indicating the number of cells, and color indicating persistence.

Line 593: Typo “on” to “one”.

Line 607: This paragraph is great, but it should come before the discussion on the effects of J, lambda and PP.

Reviewer #2: This is a comprehensive computational study on how tumours invade surrounding tissue given different physical characteristics. The paper builds a computer simulation of differing adhesion, motility and proliferation using the Cellular Potts Model. They ran 1331 different combinations of three key parameters, with 10 replicates each.

The authors identified four invasion phenotypes (distinct behavioural modes) and this paper links back to biology to refer to key tumour invasion dynamics known in the literature.

Below are the remaining concerns with the paper that the authors should address:

1) The authors should justify the rule-based classifier as the outcome of the phenotypes depends entirely on these thresholds. This could also be addressed as a sensitivity analysis or validated against manually labelled snapshots.

2) The fixed leader fraction should be explored but is labelled as future work. The leader fraction is also stated as 30% in other studies. Did the authors consider running 2 condition comparisons on either side of 25% e.g. 15 and 35% to show that this value is robust? I understand that computational resources might prevent a full run on each of these 3 (15, 25, 35%) leader fractions.

3) Proliferation is not a primary determinant of invasion mode, but it does regulate metastatic potential by controlling cluster size and persistence. Proliferation Plays a Limited Role" should be reworded to reflect this nuance.

In addition please address repeated sections in lines 234-239 and 254-258. They seem to convey the same information.

Reviewer #3: This manuscript by Akeeb et al investigates how the Cellular Potts model generates various forms of cell escape from a cellular mass representing the edge of the tumor. By varying three different model parameters, the authors formulate hypotheses of which mechanical properties of the cells are responsible for distinct patterns of cell migrations out of the tumor edge. The results are interesting and within the scope of the PLOS Computational Biology journal. I would recommend this manuscript for publications provided the critiques listed below are addressed.

1. The authors should be more precise about what is actually modeled. As the authors mentioned in the introduction about tumor invasion “… local invasion, where tumor cells breach the basement membrane and infiltrate surrounding tissues”. The presented model has no basement membrane, and no organ structure that the tumor is growing in — the lung for NSCLC; thus the title and models results should indicate that this is model of the in vitro system.

2. The invasion should be defined. How far from the egg of the tumor mass the cells must be to be considered invasive?

3. The computational model should be defined in the main text, not relegated to Supportive material.

4. In the Friedl 2012 paper, there are 8 different patters of individual/collective cell migration identified. Were all these patterns achieved by the CP model in this manuscript?

is a 3D parameter space with

5. Fig.3. — the details of these simulations are not visible, such as single cell. White background may be better for visibility of the results.

6. Fig.4A — This 3D parameter space should be drawn transparent to make the show shapes of the subspaces at the back side.

7. How the 3D parameter space in Fig.4A compares to the 3D parameter space of cell migration from Friedl 2012 paper that the authors cited?

8. Fig.4 — the majority of simulated patterns are multimodal, is this pattern observed in biology?

9. Section “Cluster formation peaks …”, nine of the ten references are to three Supplemental figures. If these figures are necessary for understanding the results, they should be included in the main text.

10. Comparison between cells invading the tissue/matrigel with CTCs is not appropriate, CTC transport is in a completely different microenvironment

11. Most of the presented results are quite obvious: invasion is dictated by migration, depends on weak adhesion, and not on proliferation. Is there any unexpected result that was generated y the model?

12. page 9. It seems that followers-only clusters are allowed. How the followers only clusters can separate from the main tumor mass, if there isn leaders in the cluster? Aren’t the followers following the leader cell?

13. Table 1 should contain parameters with physical units and references to biological literature that is a source of experimental values that agree with the values chosen for parameters.

14. How the model can be calibrated to experimental data? What should be measured experimentally to fit the J parameters?

**********

**Have the authors made all data and (if applicable) computational code underlying the findings in their manuscript fully available?**

The PLOS Data policy requires authors to make all data and code underlying the findings described in their manuscript fully available without restriction, with rare exception (please refer to the Data Availability Statement in the manuscript PDF file). The data and code should be provided as part of the manuscript or its supporting information, or deposited to a public repository. For example, in addition to summary statistics, the data points behind means, medians and variance measures should be available. If there are restrictions on publicly sharing data or code —e.g. participant privacy or use of data from a third party—those must be specified.

Reviewer #1: None

Reviewer #2: Yes

Reviewer #3: Yes

**********

PLOS authors have the option to publish the peer review history of their article (what does this mean?). If published, this will include your full peer review and any attached files.

If you choose “no”, your identity will remain anonymous but your review may still be made public.

**Do you want your identity to be public for this peer review?**  For information about this choice, including consent withdrawal, please see our Privacy Policy.

Reviewer #1: No

Reviewer #2: No

Reviewer #3: No

**Figure resubmission:**

After uploading your figures to PLOS’s NAAS tool - https://ngplosjournals.pagemajik.ai/artanalysis, NAAS will process the files provided and display the results in the "Uploaded Files" section of the page as the processing is complete. If the uploaded figures meet our requirements (or NAAS is able to fix the files to meet our requirements), the figure will be marked as "fixed" above. If NAAS is unable to fix the files, a red "failed" label will appear above. When NAAS has confirmed that the figure files meet our requirements, please download the file via the download option, and include these NAAS processed figure files when submitting your revised manuscript.

**Reproducibility:**

To enhance the reproducibility of your results, we recommend that authors of applicable studies deposit laboratory protocols in protocols.io, where a protocol can be assigned its own identifier (DOI) such that it can be cited independently in the future. Additionally, PLOS ONE offers an option to publish peer-reviewed clinical study protocols. Read more information on sharing protocols at https://plos.org/protocols?utm_medium=editorial-email&utm_source=authorletters&utm_campaign=protocols

# Author comment: Author response to Decision Letter 1

**DOI:** 10.1371/journal.pcbi.1014747.r002

28 May 2026

Attachment Submitted filename: RespondtoReviewers.pdf

# Peer review: Decision Letter 1

**DOI:** 10.1371/journal.pcbi.1014747.r003

**Authors:** Dimitrios Vavylonis, Alex Tam

16 Jul 2026

PCOMPBIOL-D-26-00250R1

Clusters, Fingers, and Singles: A Mechanical Landscape of Tumor Invasion

PLOS Computational Biology

Dear Dr. Jiang,

Thank you for submitting your manuscript to PLOS Computational Biology. After careful consideration, we feel that it has merit but does not fully meet PLOS Computational Biology's publication criteria as it currently stands. Therefore, we invite you to submit a revised version of the manuscript that addresses the points raised during the review process.

Please submit your revised manuscript by Sep 15 2026 11:59PM. If you will need more time than this to complete your revisions, please reply to this message or contact the journal office at ploscompbiol@plos.org. When you're ready to submit your revision, log on to https://www.editorialmanager.com/pcompbiol/ and select the 'Submissions Needing Revision' folder to locate your manuscript file.

Please include the following items when submitting your revised manuscript:

* A letter that responds to each point raised by the editor and reviewer(s). You should upload this letter as a separate file labeled 'Response to Reviewers'. This file does not need to include responses to formatting updates and technical items listed in the 'Journal Requirements' section below.

* A marked-up copy of your manuscript that highlights changes made to the original version. You should upload this as a separate file labeled 'Revised Manuscript with Track Changes'.

* An unmarked version of your revised paper without tracked changes. You should upload this as a separate file labeled 'Manuscript'.

If you would like to make changes to your financial disclosure, competing interests statement, or data availability statement, please make these updates within the submission form at the time of resubmission. Guidelines for resubmitting your figure files are available below the reviewer comments at the end of this letter.

As the corresponding author, your ORCID iD is verified in the submission system and will appear in the published article. PLOS supports the use of ORCID, and we encourage all coauthors to register for an ORCID iD and use it as well. Please encourage your coauthors to verify their ORCID iD within the submission system before final acceptance, as unverified ORCID iDs will not appear in the published article. Only the individual author can complete the verification step; PLOS staff cannot verify ORCID iDs on behalf of authors.

We look forward to receiving your revised manuscript.

Kind regards,

Alex Tam

Academic Editor

PLOS Computational Biology

Dimitrios Vavylonis

Section Editor

PLOS Computational Biology

**Additional Editor Comments (if provided):**

Overall, the reviewers believed that the revised manuscript did a good job of addressing the comments from the original reviews. Reviewer 1 offers a few additional comments, which I invite the authors to consider.

**Reviewers' comments:**

Reviewer's Responses to Questions

**Comments to the Authors:**

**Please note here if the review is uploaded as an attachment.**

Reviewer #1: I am pleased with the revised manuscript by Akeed and colleagues. One of my main critiques: the sequence and choice of figure panels, was fully addressed and improved the manuscript. Most of my minor concerns were also addressed by the authors in a satisfactorily way. The addition of two different baselines for leader cell fractions (15% and 35%) to show that results were not qualitatively different under those scenarios increased confidence in the results of this work.

However, in my view the authors did not fully address my second main concern: the lack of clear supporting data/figures for their claim about cluster leader fraction and persistence time. There are a few interesting results mention on the paragraph that starts on line 519, but there is no figure reference. Line 524 references panel insets, but the main figure it references is not mentioned. It is probably either figure S11 or S12, but I found these two figures very hard to read and understand. The same concern arises in the next paragraph which references figures S12 and Figure S10. I do not doubt the results, but the lack of clear figures/data to support each claim in these two paragraph weakens the paper and may compromise confidence in an otherwise very good work.

Below are a few additional notes:

Line 112: Repeated information. Better to replace that sentence with an explanation of what an MCS is.

Line 124: “Simplified” 2x

Lines 360-1. There are two mean number of finger values, are they with respect to intermediate adhesion only and strong migration only?

Lines 405-7: “The most consistent effect of PP was on the overall invasive area, which increased under high proliferation ...” I do not see this trend in Fig 3A. The PP lines seem to actually decrease.

Line 407: This sentence seems more appropriate in the figure caption.

Line 508-9: No data/figure referenced to support observation.

Lines 345-50 and 356-7: The px◯2 number carry no meaning by themselves. My suggestions are to either convert this to micrometers◯2 (which by itself also has not much meaning as it depends on how wide the simulation is) or speak in terms of fold or % increases/decreases with respect to other scenarios.

Reviewer #2: The authors have now addressed the issues raised from the initial review with extra analyses.

Reviewer #3: Thanks for addressing all my critiques and concerns. I would suggest to incorporate your answer to my comment #11 in the discussion section. I wioll leave this to your decision, but I think that the readers would appreciate a summary of what are the novel and non-intuitive findings from your research.

**********

**Have the authors made all data and (if applicable) computational code underlying the findings in their manuscript fully available?**

The PLOS Data policy requires authors to make all data and code underlying the findings described in their manuscript fully available without restriction, with rare exception (please refer to the Data Availability Statement in the manuscript PDF file). The data and code should be provided as part of the manuscript or its supporting information, or deposited to a public repository. For example, in addition to summary statistics, the data points behind means, medians and variance measures should be available. If there are restrictions on publicly sharing data or code —e.g. participant privacy or use of data from a third party—those must be specified.

Reviewer #1: Yes

Reviewer #2: Yes

Reviewer #3: Yes

**********

PLOS authors have the option to publish the peer review history of their article (what does this mean?). If published, this will include your full peer review and any attached files.

If you choose “no”, your identity will remain anonymous but your review may still be made public.

**Do you want your identity to be public for this peer review?**  For information about this choice, including consent withdrawal, please see our Privacy Policy.

Reviewer #1: No

Reviewer #2: No

Reviewer #3: No

[NOTE: If reviewer comments were submitted as an attachment file, they will be attached to this email and accessible via the submission site. Please log into your account, locate the manuscript record, and check for the action link "View Attachments". If this link does not appear, there are no attachment files.]

**Figure resubmission:**

**Reproducibility:**

To enhance the reproducibility of your results, we recommend that authors of applicable studies deposit laboratory protocols in protocols.io, where a protocol can be assigned its own identifier (DOI) such that it can be cited independently in the future. Additionally, PLOS ONE offers an option to publish peer-reviewed clinical study protocols. Read more information on sharing protocols at https://plos.org/protocols?utm_medium=editorial-email&utm_source=authorletters&utm_campaign=protocols

# Author comment: Author response to Decision Letter 2

**DOI:** 10.1371/journal.pcbi.1014747.r004

17 Aug 2026

Attachment Submitted filename: rebuttal2.pdf

# Editor report: Decision Letter 2

**DOI:** 10.1371/journal.pcbi.1014747.r005

**Authors:** Dimitrios Vavylonis, Alex Tam

20 Aug 2026

Dear Dr. Jiang,

We are pleased to inform you that your manuscript 'Clusters, Fingers, and Singles: A Mechanical Landscape of Tumor Invasion' has been provisionally accepted for publication in PLOS Computational Biology.

Before your manuscript can be formally accepted you will need to complete some formatting changes, which you will receive in a follow up email. A member of our team will be in touch with a set of requests.

Please note that your manuscript will not be scheduled for publication until you have made the required changes, so a swift response is appreciated.

IMPORTANT: The editorial review process is now complete. PLOS will only permit corrections to spelling, formatting or significant scientific errors from this point onwards. Requests for major changes, or any which affect the scientific understanding of your work, will cause delays to the publication date of your manuscript.

Should you, your institution's press office or the journal office choose to press release your paper, you will automatically be opted out of early publication. We ask that you notify us now if you or your institution is planning to press release the article. All press must be co-ordinated with PLOS.

Thank you again for supporting Open Access publishing; we are looking forward to publishing your work in PLOS Computational Biology.

Best regards,

Alex Tam

Academic Editor

PLOS Computational Biology

Dimitrios Vavylonis

Section Editor

PLOS Computational Biology

***********************************************************

Thank you for your thorough responses to the reviewer comments throughout this process.

# Editor report: Acceptance letter

**DOI:** 10.1371/journal.pcbi.1014747.r006

**Authors:** Dimitrios Vavylonis, Alex Tam

PCOMPBIOL-D-26-00250R2

Clusters, Fingers, and Singles: A Mechanical Landscape of Tumor Invasion

Dear Dr Jiang,

I am pleased to inform you that your manuscript has been formally accepted for publication in PLOS Computational Biology. Your manuscript is now with our production department and you will be notified of the publication date in due course.

The corresponding author will soon be receiving a typeset proof for review, to ensure errors have not been introduced during production. Please review the PDF proof of your manuscript carefully, as this is the last chance to correct any errors. Please note that major changes, or those which affect the scientific understanding of the work, will likely cause delays to the publication date of your manuscript.

Soon after your final files are uploaded, unless you have opted out, the early version of your manuscript will be published online. The date of the early version will be your article's publication date. The final article will be published to the same URL, and all versions of the paper will be accessible to readers.

For Research, Software, and Methods articles, you will receive an invoice from PLOS for your publication fee after your manuscript has reached the completed accept phase. If you receive an email requesting payment before acceptance or for any other service, this may be a phishing scheme. Learn how to identify phishing emails and protect your accounts at https://explore.plos.org/phishing.

Thank you again for supporting PLOS Computational Biology and open-access publishing. We are looking forward to publishing your work!

With kind regards,

Anitha Samidurai

PLOS Computational Biology | Carlyle House, Carlyle Road, Cambridge CB4 3DN | United Kingdom ploscompbiol@plos.org | Phone +44 (0) 1223-442824 | ploscompbiol.org | @PLOSCompBiol
